# Intelligent *in-silico* prioritization of antimalarial peptide candidates under explicit physicochemical windows via *de novo* CTCM-Neo generation and conformal-gated calibrated classification

**DOI:** 10.3389/fcimb.2026.1707267

**Published:** 2026-03-04

**Authors:** Muhammad Aamir, Khosro Rezaee, Maryam Saberi Anari

**Affiliations:** 1 College of Computer Science and Artificial Intelligence, Huanggang Normal University, Huanggang, Hubei, China; 2 Department of Biomedical Engineering, Meybod University, Meybod, Iran; 3 Department of Computer Engineering, Technical and Vocational University (TVU), Tehran, Iran

**Keywords:** antimalarial peptides, conformal prediction, CTCM-Neo, *de novo* design, generalization, hyperparameter sensitivity, positive–unlabeled learning

## Abstract

**Introduction:**

Malaria remains a major global health burden and motivates fast, reliable in silico prioritization of antimalarial (AM) peptide candidates. Designing such peptides is challenging due to the vast search space, scarce or noisy supervision, and potential out-of-distribution miscalibration of computational scores. Prior pipelines typically rank existing sequences rather than generate new candidates under explicit design constraints with calibrated, risk-aware decision rules.

**Methods:**

We propose a constraint-guided generate–then–classify framework. A low-data generator—an optimized variant of CTCM-Neo—proposes de novo sequences within APD3-derived windows for net charge, GRAVY, and Boman index. A frozen, temperature-scaled protein language-model classifier (ConformaX-PEP) outputs calibrated probabilities for predicted antimalarial activity and hemolysis, and a split-conformal gate with risk level α=0.1 converts these scores into accept/reject decisions at fixed operating thresholds *p*_*act*_ ≥ 0.78 and *p*_*hemo*_ ≤ 0.20.

**Results:**

On the initial 322-sequence corpus (52 AM, 200 unlabeled, 70 positive-like), a held-out evaluation achieves AUROC ≈0.93, AUPRC ≈0.80, and ECE ≈0.03, indicating strong discrimination with low calibration error prior to external testing. The method outperforms strong baselines in convergence speed and reliability. On 210 previously unseen peptides (80 AM, 130 NM), two independent runs achieve 92.86% and 93.33% accuracy with balanced precision and recall and good calibration. Hyperparameter sweeps reveal broad, stable optima, supporting reproducibility. Template-based docking with GalaxyPepDock is used strictly as a hypothesis-generating structural sanity check and does not constitute evidence of biological binding or efficacy.

**Discussion:**

Overall, the framework compresses the search space into a small, risk-bounded set of computationally prioritized candidates and provides a scalable, uncertainty-aware route for downstream experimental follow-up. All results reported here are computational, and antimalarial activity remains to be confirmed experimentally.

## 1 Introduction

Malaria continues to impose a heavy global health burden: in 2022 there were an estimated 249 million cases and 608,000 deaths, with African children bearing the greatest share. The WHO-recommended vaccines RTS,S/AS01 (2021) and R21/Matrix-M (2023) are encouraging, yet moderate efficacy, supply/coverage constraints, and the parasite’s evolutionary dynamics underscore the need for complementary tools for prevention and treatment (World Health Organization, 2023a; World Health Organization, 2023b; Centers for Disease Control and Prevention, 2024; Ageitos et al., 2025). In this context, peptides—especially antiplasmodial peptides—offer an agile, engineerable modality to help close the therapeutic gap.

Peptides are attractive because they are straightforward to synthesize, readily tunable in sequence and structure, and can be designed for multi-target action; at the same time, challenges such as *in vivo* stability, target specificity, and safety (e.g., hemolysis/toxicity) must be managed. The APD3 database and its current incarnation curate rich physicochemical patterns and bioactivity labels (including “antimalarial”), which we leverage to define design constraints (net charge, GRAVY, Boman index) and to inspire generative models (Wang et al., 2016). Recent reviews also indicate that Machine Learning (ML) can accelerate AMP discovery and optimization, although translation to robust *in vivo* efficacy still requires a staged validation pipeline (Ditz et al., 2023).

Over the past decade, largely *in-silico* pipelines have matured: Human Leukocyte Antigen (HLA)-binding prediction for AMA1/CSP epitopes with human CD8^+^ ELISpot validation exemplifies how computational filters can compress an otherwise vast experimental search space. “Reverse/multi-epitope vaccinology”—with HLA coverage, linker/adjuvant design, and docking/MD evaluation—has become a transparent, modular workflow, albeit one whose clinical translation remains limited (Lee et al., 2015; Reynisson et al., 2020; Johansson-Åkhe et al., 2020). These efforts show that well-designed filters can make the search tractable without guaranteeing protection at the bedside.

With the emergence of domain-specific benchmarks for malaria antigens/epitopes, model reproducibility and fair evaluation have improved; positive–unlabeled (PU) learning is well suited to sparsely labeled regimes and has been applied in malaria contexts (Wang et al., 2019; Chou et al., 2024a; Wistuba-Hamprecht et al., 2024). For antigen presentation, the NetMHCpan family and newer deep-learning methods substantially improve pre-filter accuracy and underpin peptide design workflows (Chaudhuri et al., 2008; van Dijk et al., 2025; Bhalerao et al., 2024). In parallel, sequence-generative models (VAE/GAN/LLM) now enable *de novo* AMP design at scale, potentially fueling the search space for antiplasmodial peptides (Ditz et al., 2023; Santos-Júnior et al., 2024; Jeong et al., 2024; Hu et al., 2025).

Despite progress, there are very few *de novo* frameworks tailored to antimalarial peptides that simultaneously integrate (a) low-data generation under APD3-derived physicochemical constraints, (b) frozen, calibrated scoring with explicit uncertainty (conformal risk), and (c) external generalization reported on truly unseen data with low dispersion. Against this backdrop, we developed an efficient model for automated generation and classification of antimalarial peptides: an optimized variant of the Competition of Tribes and Cooperation of Members algorithm (CTCM) that we called CTCM-Neo, which produces a large slate of *de-novo* peptide sequences seeded by APD antimalarials. For every candidate, we quantify similarity to APD entries—using both exact sequence identity and embedding-based distances—to construct a compact similarity profile that jointly anchors plausibility and enforces novelty (near-duplicates are penalized; distant outliers are retained only if other evidence is compelling). Finally, we predict antiplasmodial potential by combining this similarity profile with native dataset descriptors (charge, GRAVY, and Boman) inside a frozen, calibrated classifier gated by a conformal accept/reject rule, thereby prioritizing sequences that are close enough to known actives to be credible yet sufficiently distinct to represent genuine innovation.

The present work addresses these gaps through the CGDP framework, which couples CTCM-Neo to a calibrated, frozen classifier (ConformaX-PEP) within a conformal gate, while enforcing Charge/GRAVY/Boman windows as hard design constraints. Notably, relatively few methods have been proposed for generating efficient antimalarial sequences; many prior efforts rely on labor-intensive wet-lab screening rather than principled generative pipelines.

The contributions of this study are as follow: (1) We introduce a generation-screening chain (CTCM-Neo + ConformaX-PEP) with calibrated probabilities and conformal risk control; (2) we derive APD3-based design windows and apply them as hard constraints alongside explicit diversity/uniqueness control (CD-HIT ≤ 40%); (3) we conduct rigorous evaluation on held-out data and on 210 completely unseen peptides, maintaining calibration with very low run-to-run dispersion; and (4) we add standard, template-based docking as a sanity check for chemo-structural compatibility rather than as a wet-lab claim (Johansson-Åkhe et al., 2020; Wang et al., 2019).

After temperature calibration, PU-prior tuning, and selecting dropout = 0.20, the classifier attains a stable operating point (AUROC ≈ 0.93, AUPRC ≈ 0.80, ECE ≈ 0.03). Adding a conformal α = 0.10 provides ~0.91 coverage without sacrificing discrimination. On a cohort of 210 unseen sequences (80 AM/130 NM), two independent runs yield 92.86% and 93.33% accuracy with balanced precision/recall/F1 and minimal variance—evidence of robust external generalization.

Compared with rule-based immunoinformatics, our approach brings *de novo* design supported by explicit calibration and conformal risk control; compared with purely predictive ML, it emphasizes external generalization under a fixed decision threshold. On the structural side, we use GalaxyPepDock (template-based) together with standard metrics for peptide–protein docking to assess the spatial/chemical plausibility of selected poses—while emphasizing that such *in-silico* evidence does not substitute for wet-lab or *in-vivo* validation (Johansson-Åkhe et al., 2020; Periwal et al., 2024; McFadden et al., 2025).

Section 2 reviews prior work; Section 3 details our proposed method, including data curation, APD3-derived constraints, and standardization, followed by the architectures of CTCM-Neo and ConformaX-PEP with calibration/conformal gating. Sections 4 and 5 report results and statistical evaluations on internal splits (split-safe) and the 210-sequence external set. The paper concludes with a synthesis of findings and implications for future experimental validation.

## 2 Related work

Most prior work spans curated benchmarks and antigen/epitope screening, PU learning at the proteome scale, and immunoinformatics pipelines that integrate HLA binding, docking, and molecular dynamics (MD). Structure-aware predictors (Graph Neural Networks, GNNs/transformers) improve Major Histocompatibility Complex (MHC) and B-cell epitope filtering, while large-scale ML mining has expanded the Antimicrobial Peptide (AMP) search space.

Most prior work spans curated benchmarks and antigen/epitope screening, PU learning at the proteome scale, and immunoinformatics pipelines that integrate HLA binding, docking, and MD. Structure-aware predictors (Graph Neural Networks, GNNs/transformers) improve Major Histocompatibility Complex (MHC) and B-cell epitope filtering, while large-scale ML mining has expanded the AMP search space.

Ditz et al. (2023) introduced PlasmoFAB, a curated benchmark for ML-based antigen/epitope candidate prediction in P. falciparum, demonstrating that tailored datasets outperform generic localization services. By standardizing positives/negatives and publishing code/labels, they enabled reproducible ML for malaria antigen discovery. The work’s clear strength is dataset quality; a limitation is that it addresses antigen candidacy rather than direct peptide construct design.

Chou et al (2024b) implemented PU learning over multi-omics features (272 variables across ~5,400 proteins) to rank P. falciparum vaccine antigens, explicitly quantifying variable importance and stage-specific expression. The ML framework generalized beyond handcrafted filters and highlighted conserved, essential candidates less prone to immune escape. It powerfully broadens the search space; still, it requires iterative experimental validation to confirm true protectivity.

Wistuba-Hamprecht et al. (2024) trained machine-learning models on antibody profiling data to predict malaria vaccine efficacy, showing that humoral feature spaces can forecast protection and thus guide which peptide epitope sets should be emphasized in design iterations. This creates a feedback loop: peptide candidates to antibody profiles to ML efficacy predictions and to refined peptide selection. The strength is endpoint relevance (efficacy); a limitation is that models are tied to specific cohorts and vaccines.

Chaudhuri et al. (2008) introduced MalVac, an early curated resource that aggregated malarial vaccine candidates and peptide epitopes to support reverse vaccinology and algorithmic down-selection. Although not ML per se, MalVac systematized *in-silico* filters (signal peptides, localization, and antigenicity) that later pipelines automated. Its chief value was standardizing inputs; its limitation was static curation and pre-ML heuristics.

van Dijk et al. (2025) combined epitope mapping with computational screening to identify conserved human T-cell epitopes within PfCSP that are broadly recognized across HLA backgrounds, directly informing peptide selection for sporozoite-stage vaccines. Although not a pure design paper, it provides rigorous epitope-level constraints (conservation, human recognition) essential for ML-guided peptide prioritization in malaria. The strength is high-quality clinical immunology; a limitation is that *de novo* peptide generation/optimization is out of scope.

Bhalerao et al. (2024) used reverse vaccinology and epitope-prediction tools (NetCTL/NetMHCII/IEDB) to design a multi-epitope Pf construct, optimizing peptide linkers/adjuvants and modeling receptor interactions by docking and MD. Their selection criteria explicitly combine ML-predicted antigenicity, non-allergenicity, and HLA coverage to prioritize short peptides. The pipeline is transparent and replicable, though entirely *in silico*.

Santos-Júnior et al. (2024) performed a cell-level ML mining of the global microbiome (AMPSphere), predicting ~0.86 M non-redundant AMPs and validating 79/100 synthesized peptides experimentally, thereby establishing a massive search space for downstream task-specific optimization. For malaria, these sequences can be re-filtered against Pf targets (e.g., heme detoxification, invasion ligands) and human-safety predictors, then refined by GNN/transformer models. The strength is the scale and high hit rate; a limitation is that antiplasmodial activity was not the original endpoint.

Jeong et al. (2024) proposed GraphMHC, a GNN that represents MHC–peptide complexes as molecular graphs and achieved AUROC ≈ 0.92 for binding prediction, surpassing a baseline deep model. This improves the precision of peptide–HLA filtering in malaria pipelines, especially for diverse HLA repertoires. Strength: structure-aware learning; limitation: trained largely on cancer/IEDB data, so transfer to Pf antigens must be verified.

Hu et al. (2025) built deepBCE-Parasite, a transformer model for parasite B-cell epitope prediction that reached ~81% accuracy and AUC ~0.90 and experimentally validated 7/8 predicted peptides (dot blot) in a parasitic system. Though demonstrated in Fasciola, the framework is directly applicable to Pf antigens for peptide vaccine screening. The strength is experimental confirmation; the limitation is cross-species generalization still needs testing in malaria.

Periwal et al. (2024) developed an ML classifier for antiprotozoal peptides, testing 15 models and reporting a best Extra-Trees configuration with accuracy ~92% and AUC ~0.97 on an independent test set. While pan-protozoal, the trained feature representations and ranking strategy are directly useful for pre-screening antiplasmodial peptide candidates before docking/MD. The limitation is that labels aggregate protozoa broadly and may need Pf-specific fine-tuning.

Pandey et al. (2018) applied an immunoinformatics pipeline to mosquito salivary proteins, integrating B- and T-cell epitope predictions, structural filtering, disulfide “stabilization” design, and docking to design a multi-epitope subunit peptide vaccine against malaria. *In-silico* physicochemical screens and immune simulations suggested favorable antigenicity and population coverage. Advantages were end-to-end automation; constraints were lack of wet-lab efficacy.

Heide et al. (2019) systematically reviewed P. falciparum-specific CD8^+^ T-cell epitopes, consolidating experimentally confirmed peptides and contextualizing prediction tools and HLA restriction. The synthesis underpins ML feature choices (motifs, length, HLA supertypes) and helps benchmark predicted immunogenicity. A strength is breadth; a limitation is that it is retrospective rather than generative.

Schmedes et al. (2022) (while diagnostic rather than design) used decision-tree ML on multiplex antigen concentrations (HRP2, pLDH, pAldolase) from field samples to classify PCR-confirmed infection status (73%–96% accuracy) and parasite density tiers. This work illustrates how ML learns quantitative antigen signatures and supports algorithmic feature selection relevant to peptide target prioritization in surveillance-aware vaccine design. Limits include cohort size and absence of vaccine endpoints, but the ML framing is directly portable.

Mandal et al. (2025) designed a blood-stage multiepitope vaccine by combining several Pf proteins (PfPHB1/2, PfHSP70, and PfGARP) and running a standard reverse-vaccinology/ML stack (VaxiJen/ANTIGENpro for antigenicity, NetMHC/IEDB for class I/II binding, AllerTOP/ToxinPred for safety, and ClusPro/HADDOCK for docking). Molecular dynamics and immune simulations supported stable TLR engagement and balanced T- and B-cell responses *in silico*. The workflow is comprehensive and reproducible; however, it remains computational with no reported *in vivo* efficacy.

Choi and Kim (2024) introduced EpiGraph, a deep graph-attention network that fuses protein language-model embeddings with structural graphs to predict B-cell epitopes, outperforming recent baselines on independent benchmarks. For malaria peptide design, such a model can pre-rank Pf peptide segments for likely conformational B-cell recognition before synthesis. The advantage is explicit spatial context; limitations include dependence on structure quality.

Aguilera-Puga and Plisson (2024) reviewed structure-aware ML strategies for bioactive peptide discovery, arguing that incorporating structural priors (and not only sequence) improves generalization and mechanism interpretability. This perspective justifies using structure-infused models (GNNs, multimodal encoders) in malaria peptide pipelines to reduce false positives. The paper’s advantage is a rigorous synthesis; the limitation is that it is a review rather than a malaria-specific benchmark.

Wan et al. (2024) have presented a comprehensive review of ML for AMP discovery and design, synthesizing challenges in data curation, surveyed model families (supervised, generative, and reinforcement learning), benchmark design and evaluation protocols, multi-objective optimization criteria, and translational hurdles from *in-silico* candidates to experimental validation.

Santos-Júnior et al. (2024) have performed microbiome-scale mining in AMPSphere, predicting ~0.86 million non-redundant AMP sequences with neural classifiers and redundancy control, and have prospectively synthesized and tested a subset, reporting high experimental hit rates; they have released a searchable resource and accompanying code.

Jin et al. (2025) have introduced AMPGen, a diffusion-based, target-conditioned AMP generator that couples a learned activity scorer with knowledge-based filtering; they have produced *de novo* peptide candidates and have reported *in vitro* validation against selected microbial targets alongside ablation studies of the design pipeline.

Zhao et al. (2025) have proposed EBAMP, a Transformer-driven *de novo* broad-spectrum AMP framework that integrates sequence generation, multi-property prediction, and feature-based screening; they have benchmarked multiobjective peptide quality, provided large candidate libraries, and have documented case studies across several organisms.

Lin et al. (2024) have presented MalariaFlow, a deep-learning platform for antimalarial activity prediction across parasite life stages and strains using curated assay datasets; they have implemented model ensembles with standardized preprocessing and visualization utilities and have focused primarily on small-molecule inhibitors through a publicly accessible interface.

Despite this progress, a unified pipeline is missing that generates *de novo* antimalarial peptides under explicit physicochemical windows, classifies them with a frozen, calibrated model equipped with conformal acceptance, and demonstrates external generalization on truly unseen sequences with low dispersion. To our knowledge, no study offers an end-to-end generate-then-classify framework tailored to antiplasmodial peptides; our approach closes this gap by coupling a low-data generator with a rigorously calibrated classifier and a conformal gate, yielding an uncertainty-aware path from vast peptide space to credible, novel candidates validated beyond the training distribution.

## 3 Proposed model

**Figure 1:**
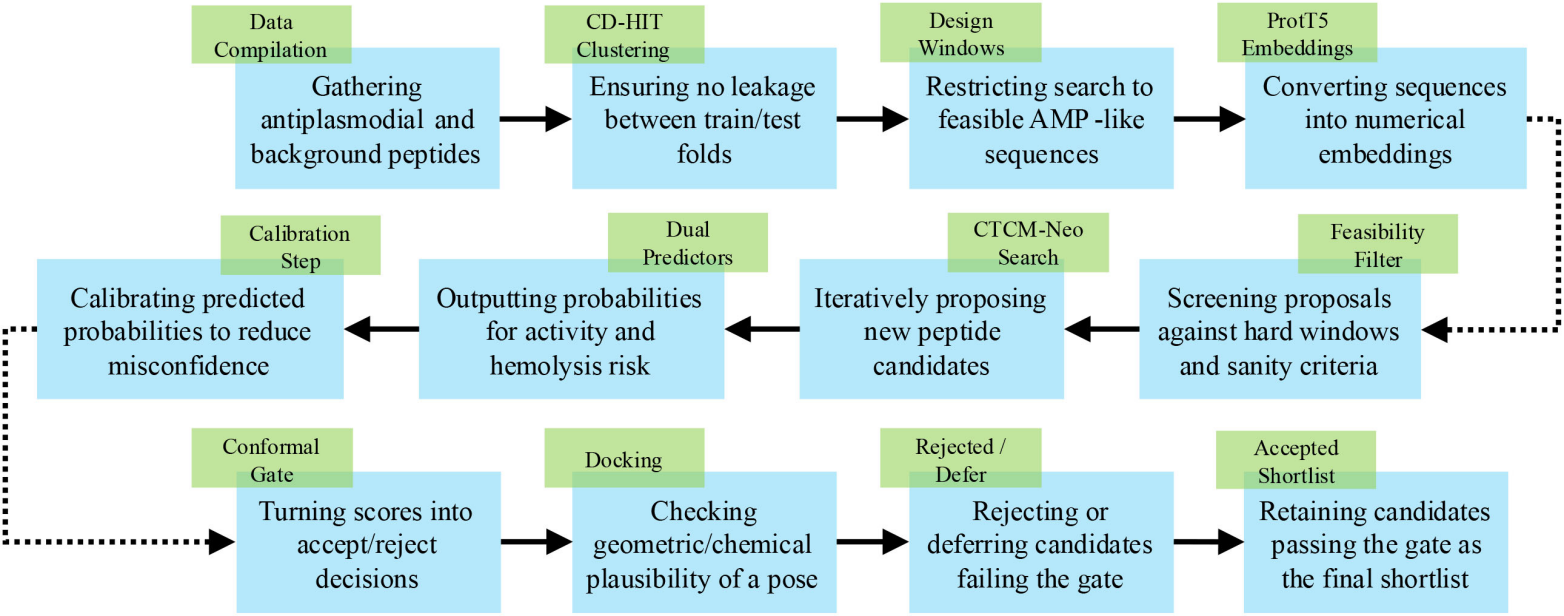
Antiplasmodial peptide prediction workflow: CTCM-Neo generation, feasibility filtering, ProtT5 embedding, dual prediction, calibration, conformal gating, and optional docking.

Here, we frame the pipeline as a generate–compare–prioritize loop anchored to APD (**Figure 1**). First, an optimized CTCM (CTCM-Neo) produces a large slate of *de novo* peptide sequences seeded by APD antimalarials. For every candidate, we then quantify its similarity to APD entries—using both exact sequence identity (e.g., Smith–Waterman/CD-HIT style measures) and embedding-based distances—to build a compact “similarity profile” that simultaneously anchors plausibility and enforces novelty (near-duplicates are penalized; distant outliers are retained only if other evidence is strong). Finally, we predict antiplasmodial potential by combining this similarity profile with the dataset’s native descriptors (charge, GRAVY, Boman) inside a frozen, calibrated classifier and a conformal acceptance gate, thereby prioritizing sequences that are close enough to known actives to be credible yet sufficiently distinct to represent genuine innovation.

### 3.1 Problem and scope

Our objective is the *de novo*, software-only design of blood-stage antimalarial peptides against *Plasmodium falciparum*, seeded by APD entries. In the seed table, the only primary descriptors available per peptide are net charge, hydrophobicity (GRAVY), and the Boman index—in addition to the raw sequence itself. We therefore treat these three as hard priors and feasibility constraints: designs are steered toward cationic, moderately amphipathic profiles within non-sticky Boman ranges, while diversity penalties prevent collapse onto a single physicochemical niche. Mechanistic claims remain computational hypotheses; for membrane-active candidates we rely on these physicochemical surrogates, and for putative PPI blockers (e.g., RH5–Basigin, AMA1–RON2) we use standard docking with decoy-based relative scoring and, if needed, brief sanity-check MD—without advancing claims in this work.

Because the base dataset exposes only charge, GRAVY, and Boman, the generator (CTCM-Neo) optimizes a multi-objective reward that (1) maximizes the classifier score for antiplasmodial activity, (2) constrains charge/GRAVY/Boman to calibrated windows derived from APD, and (3) enforces novelty via a minimum distance in embedding space. The classifier (X-PEP) consumes sequences (PLM embeddings) and optional lightweight structural surrogates we compute on the fly; however, the gatekeeping criteria at selection time intentionally depend only on what the dataset natively provides plus the classifier’s calibrated probability. Concretely, a candidate is accepted *in silico* if P(active) ≥0.80 under temperature-calibrated, conformal risk ≤10%, charge ∈ [+3, +7], GRAVY ∈ [−1.5, +0.5], Boman in a non-adhesive band, and—only for PPI-oriented designs—a docking Z-score better than decoys. We explicitly guard against overfitting by scaffold/cluster splits at ≤40% identity and report calibrated uncertainty, sensitivity analyses, and ablations (dropping structure surrogates, the diversity term, or PU learning) to keep the pipeline transparent, reproducible, and faithful to the dataset’s minimal descriptors.

### 3.2 Dataset and preprocessing

Our core corpus is the APD3 antimalarial subset (52 sequences) (APD3 database, 2025; Wang et al., 2016). For each entry, we treat the raw sequence plus its three primary physicochemical descriptors—net charge, GRAVY hydrophobicity, and the Boman index—as the canonical record and recompute all three descriptors from the sequence to remove source inconsistencies. Activity labels are drawn as positives from APD; where MIC/IC50 values are available in APD or its cited primary reports, we harmonize units to μM, apply a log_10_ transform, and retain the parasite strain/stage metadata. In the frequent absence of quantitative activity, the peptide remains a qualitative positive and is handled under PU learning. For hemolysis, when experimental values are available from APD-linked sources, we binarize against a conservative threshold (e.g., ≤5% hemolysis at 50 μM = non-hemolytic); otherwise, hemolysis contributes as a soft auxiliary target. Stability is modeled purely *in silico* as a composite proxy (predicted protease liability and helical propensity), used both as an auxiliary head in the classifier and as a penalty/reward in the generator. To construct the U-set for PU learning and to control compositional bias, we sample non-malarial APD peptides and length-/composition-matched decoys.

Sequences are standardized (IUPAC alphabet, removal of gaps/whitespace, canonical handling of simple PTMs such as amidation and N-acetylation), with entries containing unsupported noncanonical residues excluded unless explicitly modeled. We restrict the design window to 8–30 residues, deduplicate near-duplicates, and recompute + z-normalize charge/GRAVY/Boman from the sequence. Quantitative activity is harmonized to μM; if multiple assay reports exist, we store the median across strains/conditions and preserve per-strain labels (e.g., 3D7, Dd2) for stratified analyses. To prevent information leakage, we cluster the entire corpus (positives + U-set) with CD-HIT at ≤ 40% sequence identity, then perform cluster-wise train/validation/test splits so that no cluster appears in more than one split. Any augmentation (e.g., composition-preserving shuffles or conservative BLOSUM substitutions) is applied only after splitting and only to the training split. Class imbalance is addressed via sample weighting/focal loss, and decision thresholds are set under temperature-calibrated probabilities with conformal prediction for error-rate control. We report AUROC, AUPRC, MCC, and calibration metrics (Brier, ECE), alongside sensitivity analyses/ablations (dropping PU, the structure branch, or the diversity constraint) to document robustness and uphold strict, leak-free reproducibility.

### 3.3 CTCM-Neo

CTCM-Neo builds on CTCM (Chen et al., 2025b), a cooperative “tribal-chief” swarm metaheuristic (PSO-like) that we adapted for sequence design (original codebase available in our **Supplementary Material**; see **Algorithm 1**). We chose this family of optimizers because it is sample-efficient, black-box, and natively supports hard constraints, letting us enforce charge/GRAVY/Boman windows and diversity penalties during search rather than as brittle *post-hoc* filters. Unlike deep generative models (RNN/VAE/Transformers), which require large labeled corpora that do not exist for antimalarial peptides (APD has only 52 positives), CTCM-Neo can explore discrete, variable-length peptide space directly under minimal priors—well aligned with our software-only, APD-seeded setting.

Algorithm 1

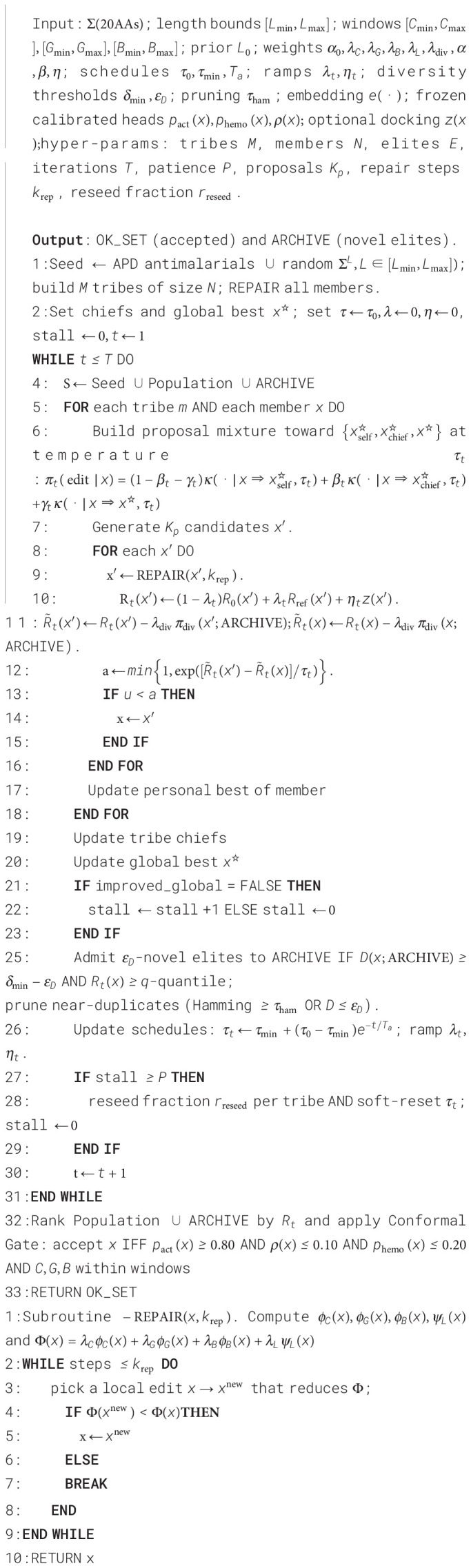

Compared with classical Genetic Algorithm (GA)/Simulated Annealing (SA) or categorical Bayesian optimization, the tribal-swarm scheme offers stronger global exploration (multi-tribe leaders) with coarse-to-fine refinement and integrates cleanly with our frozen, calibrated gate (conformal risk control) without collapsing innovation into the classifier’s bias. Relative to Reinforcement Learning Models (RLMs), CTCM-Neo avoids reward-shaping pathologies and instability, yet still accommodates multi-objective rewards (activity↑, hemolysis↓, developability↑) and optional docking-based terms for PPI blockers. In short, CTCM-Neo gives us a controllable, data-frugal, and reproducible engine for *de novo* peptide design that is particularly suited to the malaria use case, where labels are scarce and physicochemical feasibility must be honored explicitly.

#### 3.3.1 Design space and multi-objective structure

We formalize *de novo* peptide design over the discrete alphabet of the 20 canonical amino acids Σ, with variable length L∈[8,30]. The sequence space is therefore $X=\cup_{L=8}^{30}{\text{Σ}}^{L}$, and any candidate x∈X is mapped to the native descriptors our dataset reliably exposes: net charge C(x), GRAVY hydrophobicity G(x), and Boman index B(x). For diversity control we embed sequences with a fixed, pre-trained protein language model $e:X\to {\mathbb{R}}^{d}$ and define novelty as the closest embedding distance to a reference set S (e.g., APD antimalarials ∪ current population) (Equation 1):

(1) $$D(x;S)=\underset{y\in S}{\text{min}}||e(x)-e(y){\left|\right|}_{2}$$

Physicochemical feasibility is encoded as interval constraints $C_{\text{min}}\leq C(x)\leq C_{\text{max}}$, $G_{\text{min}}\leq G(x)\leq G_{\text{max}},B_{\text{min}}\leq B(x)\leq B_{\text{max}}$.

We impose them with smooth hinge penalties that vanish inside the window and grow quadratically outside; for charge (Equation 2):

(2) $$\phi_{C}(x)=1[C(x)<C_{\text{min}}]{\left(C_{\text{min}}-C(x)\right)}^{2}+1[C(x)>C_{\text{max}}]{\left(C(x)-C_{\text{max}}\right)}^{2},\text{ and analogously }\phi_{G},\phi_{B}$$

At the coarse exploration stage, we maximize a descriptor-faithful novelty objective that encourages spread in embedding space while keeping candidates feasible. With weights $\alpha_{0},\lambda_{C},\lambda_{G},\lambda_{B},\lambda_{L}\geq 0$ and a mild length regularizer $\psi_{L}(x)={\left(L(x)-L_{0}\right)}^{2}$ around prior $L_{0}$, (Equation 3)

(3) $$R_{0}(x)=\alpha_{0}D(x;S)-\lambda_{C}\phi_{C}(x)-\lambda_{G}\phi_{G}(x)-\lambda_{B}\phi_{B}(x)-\lambda_{L}\psi_{L}(x)$$

Once a frozen classifier provides calibrated activity ${\hat{p}}_{\text{act }}(x)$ and hemolysis ${\hat{p}}_{\text{hemo }}(x)$ together with conformal risk ρ(x)∈[0,1], we add a confidence-weighted activity bonus and a safety penalty (coefficients α,β≥0) (Equation 4):

(4) $$R_{\text{ref}}(x)=R_{0}(x)+\alpha {\hat{p}}_{\text{act}}(x)(1-\rho (x))-\beta {\hat{p}}_{\text{hemo}}(x)$$

For PPI-targeted designs we optionally include a docking preference z(x) (normalized negative Z-score vs. decoys) with trade-off η≥0 (Equation 5):

(5) $$R_{\text{ppi}}(x)=R_{\text{ref}}(x)+\eta z(x)$$

This formulation yields a compact, data-faithful search landscape: the novelty term D promotes exploration in a representation that captures higher-order regularities; the smooth penalties $\phi_{C},\phi_{G},\phi_{B}$ enforce biophysical windows without brittle hard clipping; the base reward $R_{0}$ enables label-free exploration; the refinement $R_{\text{ref }}$ injects calibrated evidence while discouraging hemolysis; and the optional $R_{\text{ppi }}$ biases toward plausible PPIblocking geometries. All weights are fixed *a priori* or tuned on validation clusters ( ≤40% identity) and never fit on generated samples, preserving reproducibility and preventing feedback leakage.

#### 3.3.2 Variation, constraint repair, and diversity control

We operate on variable-length sequences with a local neighborhood N(x) comprising point substitutions, single-residue insertions, and single-residue deletions at arbitrary positions, i.e (Equation 6).

(6) $$N(x)=\{x^{\left(i-a\right)}\}\cup \{x^{\left(+i,a\right)}\}\cup \{x^{\left(-i\right)}\}$$

where $x^{\left(i-a\right)}$ replaces residue i by $a\in \text{Σ},x^{\left(+i,a\right)}$ inserts a before position i, and $x^{\left(-i\right)}$ deletes residue i. To bias variation toward feasibility rather than pure random drift, we define a repair loss that aggregates the smooth penalties introduced in 3.3.1 (Equation 7),

(7) $$\text{Φ}(x)=\lambda_{C}\phi_{C}(x)+\lambda_{G}\phi_{G}(x)+\lambda_{B}\phi_{B}(x)+\lambda_{L}\psi_{L}(x)$$

and sample edits with a Boltzmann policy that prefers moves decreasing Φ (Equation 8):

(8) $$\mathbb{P}(x\to x^{\prime })\propto \text{exp}(-\kappa [\text{Φ}(x^{\prime })-\text{Φ}(x)]),\;x^{\prime }\in N(x)$$

with inverse temperature κ annealed across iterations. In practice, we exploit per-residue contributions c(a) to charge, g(a) to GRAVY, and b(a) to the Boman index to construct guided candidate sets at each locus (e.g., a∈{K,R,H} if $C(x)<C_{\text{min}};a\in \{D,E\}$ if $C(x)>C_{\text{max}}$), so that substitutions approximate a discrete, gradient-free projection back into the admissible bands while preserving local sequence context. Children produced by uniform or one-point recombination $z=C_{\theta }(x,y)$ inherit segments from two parents with mixing rate θ, and are immediately repaired by one or two greedy steps minimizing Φ before being scored by the reward of 3.3.1. Diversity is enforced both geometrically in embedding space and combinatorially at selection. Let A be an archive of accepted elites; we impose a minimum distance $\delta_{\text{min}}$ to A via a smooth penalty (Equation 9).

(9) $$\pi_{\text{div}}(x;A)=\text{max}{\left(0,\delta_{\text{min}}-D(x;A)\right)}^{2}$$

and use the diversity-regularized score $\tilde{R}(x)=R(x)-\lambda_{\text{div }}\pi_{\text{div }}(x;A)$ for ranking within tribes. Archive updates are ϵ-novelty-aware: a candidate x is admitted if $\left[D(x;A)\geq \delta_{\text{min}}-\epsilon_{D}\right]$ and R(x) exceeds a quantile threshold q of the current population, after which near-duplicates (Hamming identity ≥τ or $D\leq \epsilon_{D}$) are pruned. This coupling of repair-guided variation (Equations 6-8) with novelty-controlled selection (Equation 9) keeps the swarm inside biophysical windows while pushing outward from known APD chemotypes, yielding a stable explore-exploit balance without *post-hoc*, brittle filtering.

#### 3.3.3 Search schedule and coarse-to-fine refinement

CTCM-Neo proceeds in epochs with multiple tribes exploring in parallel. Within each tribe, candidates are updated by sampling edit proposals (substitution/insertion/deletion) from a temperature-controlled mixture kernel that pulls a sequence x toward three anchors: its own incumbent best $x_{\text{self }}^{☆}$, the tribe chief $x_{\text{chief }}^{☆}$ and the global best $x^{☆}$. Let $\kappa (\cdot |x\Rightarrow y,\tau_{t})$ denote a local edit kernel that stochastically aligns x to y and proposes discrete moves with softness governed by temperature $\tau_{t}$. The proposal distribution at iteration t is (Equation 10):

(10) $$\pi_{t}(\text{ edit }|x)=(1-\beta_{t}-\gamma_{t})\kappa (\cdot |x\Rightarrow x_{\text{self }}^{☆},\tau_{t})+|\beta_{t}\kappa (\cdot |x\Rightarrow x_{\text{chief }}^{☆},\tau_{t})+\gamma_{t}\kappa (\cdot |x\Rightarrow x^{☆},\tau_{t})$$

with exploration weights $\beta_{t},\gamma_{t}\in [0,1]$ increasing mildly over time to emphasize inter-tribe guidance as search matures. Temperature follows an exponential annealing to sharpen decisions (Equation 11):

(11) $$\tau_{t}=\tau_{\text{min}}+(\tau_{0}-\tau_{\text{min}})\text{exp}(-t/T_{a})$$

where $T_{a}$ is the annealing horizon. Early epochs thus favor broad, multi-modal exploration; late epochs concentrate proposals around chiefs and the global incumbent. The objective is interpolated coarse-to-fine. In early epochs we optimize the descriptor-faithful exploration reward $R_{0}$ (§3.3.1); after a burn-in we ramp in classifier evidence to obtain $R_{\text{ref }}$ (and, if applicable, the docking term). Writing $\lambda_{t},\eta_{t}\in [0,1]$ as monotone schedules (e.g., linear or sigmoidal ramps) (Equation 12),

(12) $$R_{t}(x)=(1-\lambda_{t})R_{0}(x)+\lambda_{t}R_{\text{ref}}(x)+\eta_{t}z(x)$$

so that the search transitions from label-free feasibility/novelty to confidence-weighted activity and optional PPI preference. Each proposed edit $x\to x^{\prime }$ is repaired case-two greedy steps reducing Φ) and then accepted with a Metropolis-style rule under the current landscape (Equation 13):

(13) $$a_{t}(x\to x^{\prime })=\text{min}\{1,\text{exp}([R_{t}(x^{\prime })-R_{t}(x)]/\tau_{t})\}$$

Stagnation triggers light re-seeding (replacing a small fraction of members with diverse samples) and a brief reset of $\tau_{t}$ within affected tribes. The final generation is ranked by $R_{t}$ and then conformally gated to enforce acceptance criteria (calibrated P (active), hemolysis risk, and descriptor windows), yielding a compact slate of high-confidence, physically plausible antimalarial peptide designs.

### 3.4 ConformaX-PEP

**Figure 2:**
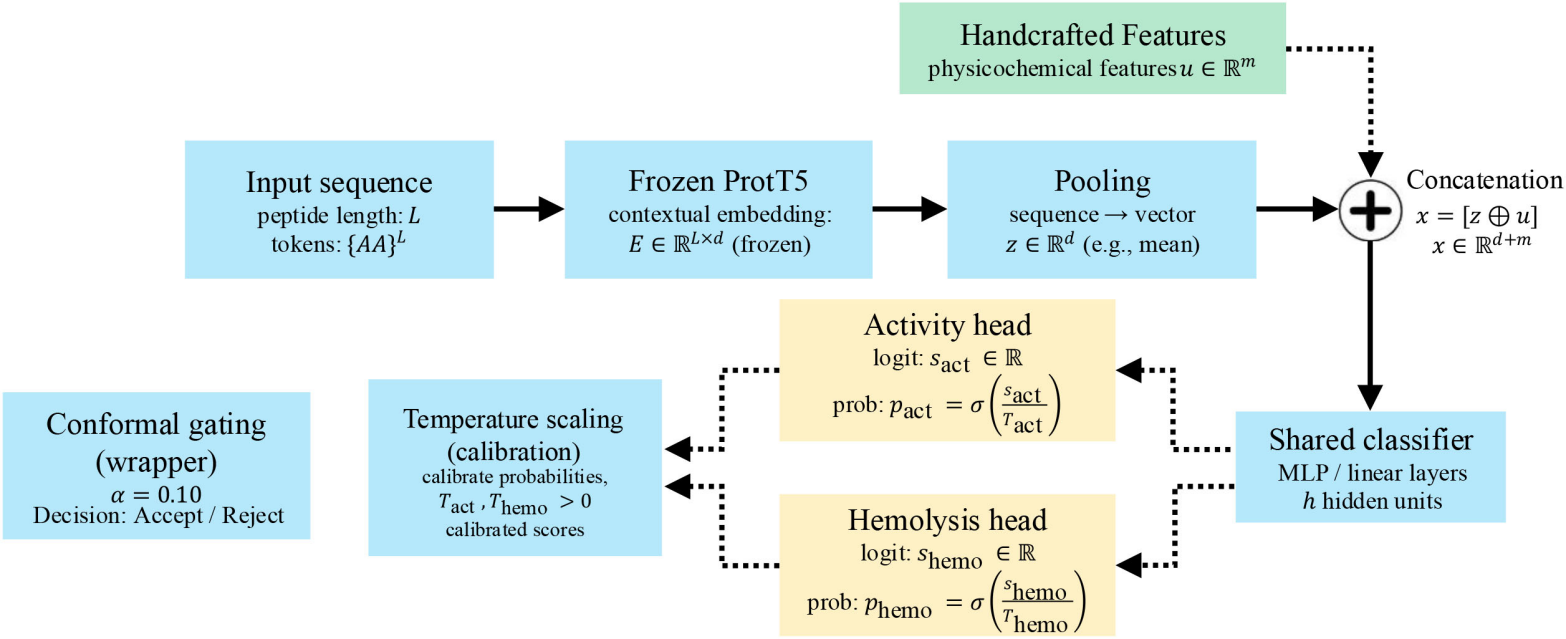
ConformaX-PEP architecture: frozen ProtT5 embeddings with feature fusion, dual heads, temperature scaling, and conformal gating.

We adopt ConformaX-PEP because APD offers few positives and only minimal, sequence-derived descriptors, making heavy end-to-end models prone to overfitting and miscalibration. ConformaX-PEP freezes a PLM backbone and adds a light fusion head over native physicochemical features (charge, GRAVY, Boman, length), yielding parameter-efficient predictions whose probabilities are calibrated via cluster-wise temperature scaling (see **Figure 2**). Coupled with a hemolysis head and wrapped in a split-conformal gate, it triages *de novo* sequences into a high-confidence, risk-bounded set, preserving novelty and feasibility while reducing downstream docking/assay burden. The classifier issues a binary decision (accept/reject as antimalarial) from the calibrated activity probability; APD similarity metrics (max sequence identity to APD actives, minimum embedding distance to APD) are not inputs to the classifier and are applied afterward in the final acceptance gate to filter near-duplicates and out-of-distribution sequences without compromising calibration. Conceptually, this design is informed by lightweight AMP predictors that combine learned sequence representations with physicochemical features and by modern calibration theory—temperature scaling for probability calibration and split-conformal prediction for distribution-free risk control—although, to our knowledge, this exact combination has not previously been instantiated for antiplasmodial peptide triage (Veltri et al., 2018; Yan et al., 2020; Hao et al., 2024; Chen et al., 2025a; Dee, 2022, Jiao et al., 2025). ConformaX-PEP uses a frozen ProtT5 backbone to produce sequence embeddings, which are pooled and optionally fused with handcrafted physicochemical descriptors ( $u\in {\mathbb{R}}^{m}$; e.g., charge, GRAVY, Boman, and length) before a lightweight shared classifier. Two heads output logits for activity and hemolysis, which are converted to calibrated probabilities via temperature scaling and then passed to a split-conformal gate ( α=0.10) to produce a risk-controlled accept/reject decision for triaging *de novo* peptides.

#### 3.4.1 Inputs and representations

Given a peptide $x=a_{1}\ldots a_{L}$ over the 20-AA alphabet, we retain only sequence and three native, sequence-derived descriptors. We bundle them as (Equation 14):

(14) $$d(x)=[\begin{array}{c} C(x)\\ G(x)\\ B(x)\\ L \end{array}],\text{ with }C(x)=\sum_{t=1}^{L}c(a_{t}),G(x)=\frac{1}{L}\sum_{t=1}^{L}g(a_{t}),B(x)=\frac{1}{L}\sum_{t=1}^{L}b(a_{t})$$

where c,g,b are per-residue charge, hydropathy (GRAVY), and Boman contributions (all computed from the sequence). A frozen protein-language model (PLM; e.g., ESM2/ProtT5) maps tokens to hidden states $h_{t}\in {\mathbb{R}}^{d}$. We form length-aware attention weights (Equation 15):

(15) $$\alpha_{t}=\frac{\text{exp}(q^{⊤}h_{t}+\gamma t/L)}{\sum_{s=1}^{L}\text{exp}(q^{⊤}h_{s}+\gamma s/L)},\;t=1,\ldots ,L,$$

and obtain the pooled PLM embedding (Equation 16):

(16) $$\bar{e}(x)=\sum_{t=1}^{L}\alpha_{t}h_{t}\in {\mathbb{R}}^{d}$$

As optional structural surrogates computable on the fly (no 3D structure needed), we predict helical propensity and protease liability from the frozen states (Equation 17):

(17) $$s(x)=[\begin{array}{c} H(x)\\ P(x) \end{array}]=[\begin{array}{c} \frac{1}{L}\sum_{t=1}^{L}\sigma (w_{H}^{⊤}h_{t})\\ \frac{1}{L}\sum_{t=1}^{L}\sigma (w_{P}^{⊤}h_{t}) \end{array}]$$

with logistic σ(·) and lightweight heads $w_{H},w_{P}$. A compact feature head encodes descriptors and fuses all signals into a single representation used by the classifier (Equation 18):

(18) $$z(x)=\text{LN}(W_{f}[\bar{e}(x)⊕\phi (W_{d}\tilde{d}(x)+b_{d});s(x)]+b_{f}),\;\tilde{d}(x)=\frac{d(x)-\mu }{\sigma }$$

where ⊕ denotes concatenation, ϕ is a small MLP nonlinearity, $\tilde{d}$ is z-scored descriptors, and LN is layer normalization. Here, L length (8-30), γ length-awareness coefficient, q pooling query, $W_{d},W_{f},b_{d},b_{f}$ trainable fusion parameters (PLM weights remain frozen).

#### 3.4.2 Architecture

The network has two branches with a light fusion block. The PLM branch keeps the language model frozen and projects token states through a tiny MLP with residual-norm, producing refined tokens ${\tilde{h}}_{t}$ (Equation 19):

(19) $${\tilde{h}}_{t}=\text{LN}(h_{t})+{\text{MLP}}_{p}(\text{LN}(h_{t})),\;t=1,\ldots ,L$$

The PhysChem branch encodes the standardized native descriptors $\tilde{d}(x)={\left[C(x),G(x),B(x),L\right]}^{⊤}$ with a very small MLP, yielding a context vector r (Equation 2):

(20) $$r={\text{MLP}}_{c}(\tilde{d}(x))$$

A single cross-attention block lets the token sequence attend to r. With learnable Q,K,V (Equation 21),

(21) $$\alpha_{t}=\frac{\text{exp}({\left(Q{\tilde{h}}_{t}\right)}^{⊤}Kr/\sqrt{d})}{\sum_{s=1}^{L}\text{exp}({\left(Q{\tilde{h}}_{s}\right)}^{⊤}Kr/\sqrt{d})},\;c=\sum_{t=1}^{L}\alpha_{t}V{\tilde{h}}_{t},\;z=[\text{Pool}({\tilde{h}}_{1:L});c;r]$$

Finally, multi-task heads map the fused representation z to calibrated probabilities for activity and hemolysis (Equation 22):

(22) $$p_{\text{act }}(x)=\sigma (w_{A}^{⊤}z+b_{A}),\;p_{\text{hemo }}(x)=\sigma (w_{H}^{⊤}z+b_{H})$$

Only the small projection/attention/heads are trainable (parameter-efficient; PLM is frozen, and LoRA/IA^3^ can be used inside Q,K,V if desired).

#### 3.4.3 Training objective

Let $g_{\text{act }}(x)$ be the activity logit and $p_{\text{act }}(x)=\sigma (g_{\text{act }}(x))$. With class-prior $\pi_{p}$ and logistic losses $\ell_{+}(g)=\text{log}(1+{\text{e}}^{-g}),\ell_{-}(g)=\text{log}(1+{\text{e}}^{g})$, the non-negative PU risk over positives P and unlabeled U for Head-A is (Equation 23):

(23) $$L_{\text{act }}=\underset{\text{positive risk}}{\underset{︸}{\pi_{p}E_{x∼P}[\ell_{+}(g_{\text{act }}(x))]}}+\text{max\{}0,\underset{\text{unlabeled as negative}}{\underset{︸}{E_{x∼U}[\ell_{-}(g_{\text{act }}(x))]}}-\pi_{p}E_{x∼P}[\ell_{-}(g_{\text{act }}(x))]\}$$

For hemolysis Head-H with targets $y_{h}\in \{0,1\}$ and prediction $p_{\text{hemo }}(x)$, we use focal-BCE (to handle imbalance/outliers) (Equation 24):

(24) $$L_{\text{hemo }}=-E[\alpha_{h}{\left(1-p_{\text{hemo }}\right)}^{\gamma }y_{h}\text{log}p_{\text{hemo }}+(1-\alpha_{h})p_{\text{hemo }}^{\gamma }(1-y_{h})\text{log}(1-p_{\text{hemo }})]$$

To stabilize under small data and enforce invariance to mild augmentations (token noise/shuffle-preserving edits), we add consistency regularization over both heads $f(x)=[p_{\text{act }}(x),p_{\text{hemo }}(x)]$ (Equation 25):

(25) $$L_{\text{cons }}=E_{x,\tilde{x}∼\text{Aug}(x)}||f(x)-f(\tilde{x}){\left|\right|}_{2}^{2}$$

The overall objective (with L2 weight decay and calibration awareness via a held-out Brier term) is (Equation 26):

(26) $$L=w_{A}L_{\text{act }}+w_{H}L_{\text{hemo }}+w_{B}{\text{ Brier }}_{\text{val }}+\lambda ||\theta {\left|\right|}_{2}^{2}$$

Calibration. After training, we apply temperature scaling on the validation split (cluster-wise) to obtain $p_{\text{act }}^{\left(T\right)}(x)=\sigma (g_{\text{act }}(x)/T)$ used at inference and by the conformal gate; network weights remain frozen.

#### 3.4.4 Calibration and conformal risk control

We first calibrate the activity head by cluster-wise temperature scaling on a held-out validation split built with ≤40% identity (to reduce ECE without changing weights). Let $g_{\text{act }}(x)$ be the pre-softmax logit and $p_{\text{act }}^{\left(T\right)}(x)=\sigma (g_{\text{act }}(x)/T)$ the temperature-scaled probability; the temperature for cluster c is chosen by minimizing the Brier loss (Equation 27):

(27) $$T_{c}^{☆}=\text{arg}\underset{T>0}{\text{min}}\frac{1}{\left|V_{c}\right|}\sum_{(x,y)\in V_{c}}{\left(p_{\text{act}}^{\left(T\right)}(x)-y\right)}^{2},p_{\text{act}}^{\left(T\right)}(x)=\sigma (g_{\text{act}}(x)/T)$$

For split-conformal prediction, we compute nonconformity scores on a separate calibration split C as $s_{i}=1-p_{\text{act }}^{\left(T\right)}(x_{i})$ and set the acceptance threshold at the (1−α)-quantile (Equation 28):

(28) $$\tau_{\alpha }={\text{ Quantile }}_{1-\alpha }(\{s_{i}:x_{i}\in C\}),\;s(x)=1-p_{\text{act }}^{\left(T\right)}(x)$$

At inference, the frozen gate accepts a candidate if and only if calibrated activity is high, conformal risk is small, hemolysis risk is low, and native descriptors lie within their windows (Equation 29):

(29) $$Accept(x)=1[p_{\text{act }}^{\left(T\right)}(x)\geq \theta_{\text{act }}\wedge s(x)\leq \tau_{\alpha }\wedge p_{\text{hemo }}(x)\leq \theta_{\text{hemo }}\wedge (C(x),G(x),B(x))\in \text{ windows }]$$

By default, we use $\theta_{\text{act }}=0.80,\alpha =0.10$ (i.e., 10% error-rate control), and $\theta_{\text{hemo }}=0.20$. This yields risk-bounded acceptance with calibrated probabilities, while keeping the classifier weights strictly frozen.

#### 3.4.5 Interpretability and sanity checks

We attribute predictions to individual residues with Integrated Gradients (IG) over the frozen PLM inputs, producing per-token saliency maps that highlight motifs and positions most responsible for $p_{\text{act }}$ and $p_{\text{hemo }}$. IG is computed along a straight-line path from a neutral baseline (e.g., uniform/UNK embedding) to the true embedding, then aggregated across embedding dimensions to yield a score per residue; we visualize these as heatmaps and report sequence-level summaries (top-k residues, span-wise attributions). To avoid spurious attention, we assess stability (saliency should be consistent under synonymous perturbations and small shuffles that preserve descriptors) and sparsity (a small subset of residues should dominate the signal). Aggregating IG across the test split further reveals global, model-preferred patterns (e.g., cationic amphipathic motifs) and helps verify that the classifier’s evidence source aligns with biophysical priors rather than idiosyncratic tokens.

Complementarily, we probe descriptor sensitivity and counterfactuals. Sensitivity curves sweep each native descriptor in isolation—computing Δp/ΔC,Δp/ΔG,Δp/ΔB by minimal residue edits that adjust charge, GRAVY, or Boman inside their admissible windows—to test directional consistency (e.g., moderate increases in positive charge should not decrease $p_{\text{act }}$ for membrane-active candidates). For counterfactuals, we perform constrained single-residue edits (substitution/insert/delete) that preserve length bounds and windows, then report $\text{Δ}p_{\text{act }},\text{Δ}p_{\text{hemo }}$ and the IG shift; candidates whose acceptance flips under tiny, window-compliant changes are flagged as borderline and down-prioritized. Together, IG heatmaps, descriptor-response plots, and constrained counterfactual deltas provide a compact, auditable check that ConformaX-PEP’s decisions are mechanistically plausible, calibrated, and robust to nuisance variation.

## 4 Results

We evaluate the proposed Conformal-Gated *De Novo* Peptide Discovery (CGDP) framework—comprising the CTCM-Neo generator and the frozen, calibrated ConformaX-PEP classifier—for *in-silico* generation and risk-controlled prioritization of predicted antimalarial peptide candidates (not experimental validation).

### 4.1 Dataset description

The working corpus comprises three components totaling 322 peptide sequences derived from the Antimicrobial Peptide Database (APD3/APD) (Wang et al., 2016). Because APD3 is actively maintained, we ensure transparency and reproducibility by explicitly documenting the dataset provenance, operational selection criteria, and the PU-learning prior policy in **Supplementary Table S1**, and by documenting the fixed-after-validation protocol for PU learning, operating thresholds, and conformal risk control in **Supplementary Table S2**. In addition, we report an explicit physicochemical constraint-compliance view for the representative *in-silico* candidates (**Table 1**) in **Supplementary Table S3**, including per-candidate length/charge/GRAVY/Boman flags and a consolidated constraints indicator.

**Table 1:** Representative predicted antimalarial peptide candidates (*in silico*) from CTCM-Neo with nearest APD3 identity.

No.	Peptide sequence	Nearest APD3/identity (%)	Net charge	GRAVY	Hydrophobic ratio (%)	Boman (kcal/mol)	Activity tags (from APD3 notes)
1	GWINEEKICKKIDERMGNTVLGGMAKAIVHKMAKNEFQCMANMDMLGNCEKHCQTSGEKGYCHGTKCKCGTPLSY	AP01792/98.67	+3.75	−0.56	37	—	Antimalarial; Antiparasitic; Broad-spectrum (Gram+/Gram−)
2	AQEPVKGPVSIKPGSCPIILIRCAMLNPPNRCLKDTDCPGIKKCCEGSCGMACFVPQ	AP01580/98.25	+3	0.071929824561404	44	—	Antimalarial; Antiparasitic; Antiviral; Antifungal; Anti-HIV; Enzyme inhibitor
3	GVIPGQKQCIALKGVCRDKLCSTLDDTIGIQNEGKKCCRRWWILEPYPTPVPKGKSP	AP02927/98.25	+5	−0.46491228070175	35	1.51	Antimalarial; Antiparasitic; Anti-inflammatory; Antibiofilm; Broad-spectrum
4	EAEEDGDLQCLCVKTTSQVRPRHITSLEVIKAGPHCPTAQLIATLKNGRKICFDLQAPLYKKIIKKLLES	AP02158/98.57	+3.5	−0.22571428571429	40	1.49	Antimalarial; Antiparasitic
5	HRHQGPIFDTAPSPFNPNQPRPGPIY	AP00171/96.15	+1.5	−1.2038461538462	19	2.26	Antimalarial; Antiparasitic; Anti-Gram+; Antifungal
6	FLSLIPHAINAVRAIAKHN	AP00546/94.74	+2.5	0.58421052631579	58	—	Antimalarial; Anti-Gram+
7	KWKLFKKIGIGAKLKVLT	AP04781/94.44	+6	0.18888888888889	50	−0.09	Antimalarial; Antiparasitic; Broad-spectrum (Gram+/Gram−)

**Table 2:** Summary of the dataset composition, including positives, unlabeled, and decoys, with key statistics on sequence length, descriptors, and splits.

Design constraints (generation)	Charge [+3,+7]; GRAVY [−1.5,+0.5]; Boman ≤1.5kcal/mol	Applied during generation (no relabeling)
Deduplication	Yes	Exact-sequence deduplication prior to splitting
Split strategy	Train 80%/Val 10%/Test 10%	Cluster-level split to prevent leakage
Clustering tool	CD-HIT	≤40% identity; whole clusters kept intact (Li and Godzik, 2006; Fu et al., 2012)
PU learning setting	P + U	PU classification with unlabeled pool potentially containing unknown positives (Elkan and Noto, 2008; Bekker and Davis, 2020)
PU prior π (activity)	0.22	Determined on validation clusters only and fixed thereafter (Elkan and Noto, 2008; Bekker and Davis, 2020)
Robust PU risk reference	nnPU	Non-negative PU risk estimator reference (Kiryo et al., 2017)
Evaluation scope	Held-out positives + unlabeled background	Generated positive-like excluded from final metrics
Design constraints (generation)	Charge [+3,+7]; GRAVY [−1.5,+0.5]; Boman ≤1.5kcal/mol	Applied during generation (no relabeling)
Deduplication	Yes	Exact-sequence deduplication prior to splitting
Split strategy	Train 80%/Val 10%/Test 10%	Cluster-level split to prevent leakage
Clustering tool	CD-HIT	≤40% identity; whole clusters kept intact (Li and Godzik, 2006; Fu et al., 2012)
PU learning setting	P + U	PU classification with unlabeled pool potentially containing unknown positives (Elkan and Noto, 2008; Bekker and Davis, 2020)
PU prior π (activity)	0.22	Determined on validation clusters only and fixed thereafter (Elkan and Noto, 2008; Bekker and Davis, 2020)
Robust PU risk reference	nnPU	Non-negative PU risk estimator reference (Kiryo et al., 2017)
Evaluation scope	Held-out positives + unlabeled background	Generated positive-like excluded from final metrics

1. APD3 positives (P; *n* = 52). The positive set contains experimentally validated antimalarial peptides curated from APD3 with reported activity against *Plasmodium* spp. (primarily *P. falciparum*—including strains 3D7, Dd2, and K1 where annotated—and *P. berghei*), as recorded in APD3 fields/notes (Wang et al., 2016). These APD3 entries constitute the only experimentally validated sequences used in this study; all sequences generated or prioritized by our pipeline remain *in-silico* candidates and were not experimentally tested here. The operational definition used to construct the positive set (including length filtering to 8–30 aa and deduplication) is specified in **Supplementary Table S1**.
2. Generated positive-like homologs (P_like; *n* = 70; calibration/analysis only). We constructed an auxiliary “positive-like” set of *de novo* sequences generated prior to the main CGDP evaluation and filtered to be high-homology neighbors of the APD3 positive set (nearest-positive matching). This set is computationally derived and not experimentally validated. It is used only for calibration/analysis (e.g., inspecting score distributions and calibration behavior) and is excluded from all final test-set metrics to avoid circularity. The operational policy governing this set (including its exclusion from final metrics) is documented in **Supplementary Table S1**.

3. Unlabeled background (U; non-malarial AMPs; *n* = 200). The unlabeled pool U was sampled from APD3 antimicrobial peptides with no malaria/Plasmodium/antimalarial/antiparasitic mention after keyword screening (Wang et al., 2016). Concretely, we applied a case-insensitive exclusion screen over APD name/notes/target fields using malaria-related terms (including: malaria, antimalarial, antiparasitic, Plasmodium, falciparum, berghei, vivax, ovale, knowlesi), retained peptides annotated for non-malarial antimicrobial activities, deduplicated sequences, filtered to 8–30 aa, and randomly sampled to *n* = 200 using a fixed seed. This design yields a challenging antimicrobial background aligned with the PU setting, where U may contain a small unknown fraction of positives (Elkan and Noto, 2008; Bekker and Davis, 2020). The operational definition of U and the keyword screen are specified in **Supplementary Table S1**.
4. Homology control and split protocol (non-leakage). To prevent sequence-similarity leakage, we performed cluster-level splitting using CD-HIT at ≤40% identity and assigned whole clusters to Train/Val/Test (80/10/10), ensuring no cluster appears in more than one split (Li and Godzik, 2006; Fu et al., 2012). This homology-control policy is explicitly documented in **Supplementary Table S1**.
5. PU-learning class prior (pi) and fixed-after-validation policy. For the activity head trained under PU learning, we treat the class prior pi as an operational prior estimated on validation clusters only and then fixed for all subsequent training and reporting. In this study, the value used throughout is pi = 0.22. The complete fixed-after-validation protocol—including the grid/range used to select pi, the selection objective (MCC/balanced accuracy at FPR ≈ 5% under calibration + gate), and the fact that pi is not re-estimated on the test split or the external evaluation—is documented in **Supplementary Table S2** (Elkan and Noto, 2008; Bekker and Davis, 2020). For robustness in PU risk estimation, we reference the non-negative PU risk formulation (nnPU) as a standard safeguard in PU learning (Kiryo et al., 2017). **Supplementary Table S2** also documents the fixed operating thresholds (p_act ≥ 0.78; p_hemo ≤ 0.20) and the conformal risk level (alpha = 0.10) used for accept/reject decisions.
6. Explicit physicochemical constraint-compliance reporting for representative candidates. During generation and candidate admission, we enforce explicit physicochemical windows (net charge, GRAVY, and Boman index) and apply the conformal gate at alpha = 0.10 under fixed operating thresholds. To improve transparency and prevent ambiguity, **Supplementary Table S3** provides a constraint-compliance view of the representative candidate set reported in **Table 1**, including per-candidate flags for length, charge, GRAVY, and Boman compliance, a consolidated Constraints_ok indicator, and brief notes for any violations or missing descriptor values.

### 4.2 Computational setup

We work with three components totaling 322 sequences (see §4.1): (i) 52 experimentally validated antimalarial peptides from APD3, (ii) 70 *de novo* “positive-like” homologs generated prior to the main evaluation and used only for calibration/analysis (never for final test metrics), and (iii) 200 non-malarial AMPs used as an unlabeled background for PU-style learning. Sequences were deduplicated, restricted to 8–30 residues, and non-canonical tokens removed. Generator-time physicochemical windows were enforced during proposal—net charge ∈ [+3,+7], GRAVY ∈ [−1.5,+0.5], Boman ≤ 1.5 kcal/mol—without altering labels (**Table 2**).

To prevent leakage, we performed cluster-level splits with CD-HIT at ≤40% identity, allocating whole clusters to Train/Val/Test (80/10/10); generated positive-like sequences inherit the cluster of their closest APD3 seed. All generalization metrics are reported only on the held-out APD3 positives plus the non-malarial background from the test split. The CTCM-Neo generator is a constrained evolutionary search over sequence space with edit operators (sub/ins/del), Metropolis-style acceptance under an annealed temperature schedule, a constraint-repair step for C/G/B windows, and an elite archive with a minimum embedding-distance rule to sustain diversity. We tuned hyperparameters via BOHB on validation clusters, optimizing for conformal-gate pass rate and novelty (embedding distance to nearest APD3 positive); budget was ≤50k proposals per run (early stop after 1k non-improving proposals), across 5 independent seeds whose archives were merged. **Table 3** summarizes ranges and the selected configuration. Sensitivity analyses showed the diversity penalty λ_div_ to be the primary lever on novelty: −50% λ_div_ led to mode collapse toward APD3-like motifs, while +50% depressed acceptance rates; a higher initial temperature T_0_ boosted exploration but required a sufficiently low T_min_ to avoid constraint violations at late stages. Unbalanced insertion/deletion probabilities skewed length distributions; the chosen 0.125/0.125 stabilized lengths within the 8–30 window.

**Table 3:** CTCM-Neo hyperparameters (search ranges and selected values).

Hyperparameter	Search range	Final (used)	Description
Population size P	{64, 96, 128, 192}	128	Balances diversity vs. compute
Elite archive size E	{8, 12, 16, 24}	16	Min embedding-distance ≥0.75
Substitution prob p_sub_	0.5-0.9	0.75	Remaining mass split across ins/del
Insertion prob p_ins_	0.05-0.25	0.125	Stabilizes lengths
Deletion prob p_del_	0.05-0.25	0.125	Stabilizes lengths
Max edit span	{1,2,3}	2	Edits per move
Repair weights (w_C_,w_G_,w_B_)	each 0.5-3.0	(2.0, 1.5, 1.0)	Penalties for C/G/B violations
Diversity penalty λ_div_	0.0-2.0	1.0	Key novelty control (see text)
Embedding model	{ESM2-650M, ProtT5-XLU50}	ProtT5-XL-U50	Frozen; distance only
Acceptance temp $T_{0}\to$ T_min_	${\text{T}}_{0}\in [1.0,3.0]$, T_min_ ∈[0.02,0.20]	2.0→0.05	Exponential decay with γ
Temperature decay γ	0.90-0.98 per epoch	0.95	–
Metropolis scale k	0.5-3.0	1.0	Score normalization
Proposals/run (max)	{20k, 35k, 50k, 75k}	50k	Early stop if no-improve =1k
No-improve patience	{300, 600, 1000}	1000	Measured in proposals
Seeded runs	{3,5}	5	Archives merged

ConformaX-PEP is a frozen-PLM classifier (ProtT5-XL-U50 embeddings, mean-pooled) concatenated with native descriptors (net charge, GRAVY, Boman, length) and two light MLP heads: p_act_ (antimalarial activity; PU-risk loss with prior π estimated on validation clusters) and p_hemo (hemolysis; focal-BCE). We trained the heads with AdamW and a cosine schedule with warm-up; applied temperature scaling on validation for calibration; and then enforced a conformal prediction gate with risk level α=0.10 alongside operational thresholds p_act_ ≥0.80 and p_hemo_ ≤0.20. **Table 4** details the search space and chosen values.

**Table 4:** ConformaX-PEP classifier hyperparameters (search and selected).

Hyperparameter	Search range	Final (used)	Description
PLM encoder	{ESM2, ProtT5-XL-U50}	ProtT5-XL-U50 (frozen)	Mean-pooled embeddings
Head hidden dim	{128, 256, 384}	256	Two MLP heads (act/hemo)
# MLP layers/head	{1,2}	2	ReLU + Dropout
Dropout (heads)	0.0-0.5	0.20	Aids calibration
Optimizer	{AdamW}	AdamW	Heads only
Learning rate	1e−5−5e−3 (log)	1.0e-3	See sensitivity
Weight decay	1e−6−1e−2	5.0e−5	–
Batch size	{32, 64, 128}	64	–
Scheduler	{Cosine, One-Cycle}	Cosine	Warm-up ratio 0.05
Epochs (max)	40-150	90	Early stop ~50-70
PU prior π (activity)	0.10-0.35	0.22	Estimated on Val; fixed thereafter
Activity loss	{PU-risk, BCE}	PU-risk	For label scarcity
Hemolysis loss	{BCE, focal γ∈[1,2]}	focal γ=1.5	Rare-positive handling
Temp scaling $T^{*}$	≥1 (free)	learned on Val	Improves ECE/Brier
Class weighting (hemo)	{off, auto}	auto	By class ratio
Conformal risk a	0.05-0.20	0.10	With thresholds p_act_ ≥0.80, p_hemo_ ≤0.20

In sensitivity tests, learning rates >2e−3 quickly overfit small clusters, while <5e−4 slowed convergence; π shifts of ±0.05 moved MCC by ∼0.02−0.04, so π was fixed post-estimation; dropout <0.1 worsened ECE, >0.4 hurt accuracy; increasing focal- γ above 1.5 improved recall of rare hemolysis cases but raised false positives slightly. Tightening p_act_ from 0.80→0.85 typically reduced acceptance by ∼10%−15% with higher precision, while α<0.10 yielded overly conservative (sometimes empty) conformal sets.

### 4.3 Model performance analysis

We evaluate CGDP along two complementary axes: *de novo* generation with CTCM-Neo and predicted activity classification with the calibrated ConformaX-PEP head. Metrics are reported on the held-out test split under cluster non-leakage (CD-HIT ≤40% identity); unless noted, operating settings are α = 0.10, $p_{\text{act }}$= 0.78, and $p_{\text{homo }}$= 0.20. Generation results emphasize yield, constraint compliance, uniqueness, and APD3 similarity; classification results cover discrimination, calibration, and conformal coverage.

#### 4.3.1 CTCM-Neo generation metrics

Within CGDP, the CTCM-Neo generator is tuned to explore sequence space aggressively while enforcing physicochemical and safety gates before admission to the accepted set.

In this antimalarial setting, where realistic design windows (length, charge, hydrophobicity) and predicted hemolysis constraints (model-based) are non-negotiable, CTCM-Neo targets-controlled exploration: the model proposes broadly, but only sequences that satisfy strict filters and pass conformal risk control (α = 0.10) are retained. The resulting yield is intentionally conservative, prioritizing biochemical plausibility and downstream viability over raw throughput.

**Table 5** summarizes a compact quality profile for the accepted set. Despite an acceptance rate of 2.5% (PPA ≈ 40), compliance with the design windows is near-saturated: 99% of sequences fall in the [8, 30] aa length range, 96% meet the net-charge [+3, +7] criterion, and 97% satisfy the GRAVY [−1.5, +0.5] window, which indicates that the generator is targeting AMP-like physicochemistry rather than sampling indiscriminately.

**Table 5:** Core generation metrics for CTCM-Neo on the accepted set (α = 0.10, test split), summarizing yield, constraint compliance, and uniqueness in the *de novo* generation and *in-silico* prioritization of novel predicted antimalarial peptide candidates.

Metric	What it means (one line)	How it’s computed	Direction	Value
Acceptance rate (%)	Fraction of proposals that pass all gates and constraints	100 × (accepted ÷ proposed)	—	2.5
PPA (proposals per accept)	Proposals needed to obtain one accepted peptide	proposed ÷ accepted = 100 ÷ Acceptance%	↓	40
Coverage — Length in [8, 30] (%)	Compliance with target length window	100 × count (length ∈ [8,30]) ÷ accepted	↑	99
Coverage — Net charge in [+3, +7] (%)	Compliance with net-charge window	100 × count (charge ∈ [+3, +7]) ÷ accepted	↑	96
Coverage — GRAVY in [−1.5, +0.5] (%)	Compliance with hydrophobicity window	100 × count (GRAVY ∈ [−1.5,+0.5]) ÷ accepted	↑	97
Coverage — Boman ≤ 1.5 (%)	Fraction with favorable binding propensity	100 × count (Boman ≤ 1.5) ÷ accepted	↑	95
Uniqueness after dedup (%)	Share of non-redundant sequences after clustering	100 × unique (CD-HIT identity ≤ 40%) ÷ accepted	↑	98

In addition, 95% meet Boman ≤ 1.5, indicating favorable binding propensity without excessive promiscuity. Post-deduplication uniqueness reaches 98% (CD-HIT ≤ 40% identity), confirming that accepted sequences are diverse rather than minor variants. Taken together, these results show that CTCM-Neo delivers high-quality, non-redundant peptide candidates tailored to antimalarial design constraints, which is precisely the bias desired before experimental triage.

**Figure 3:**
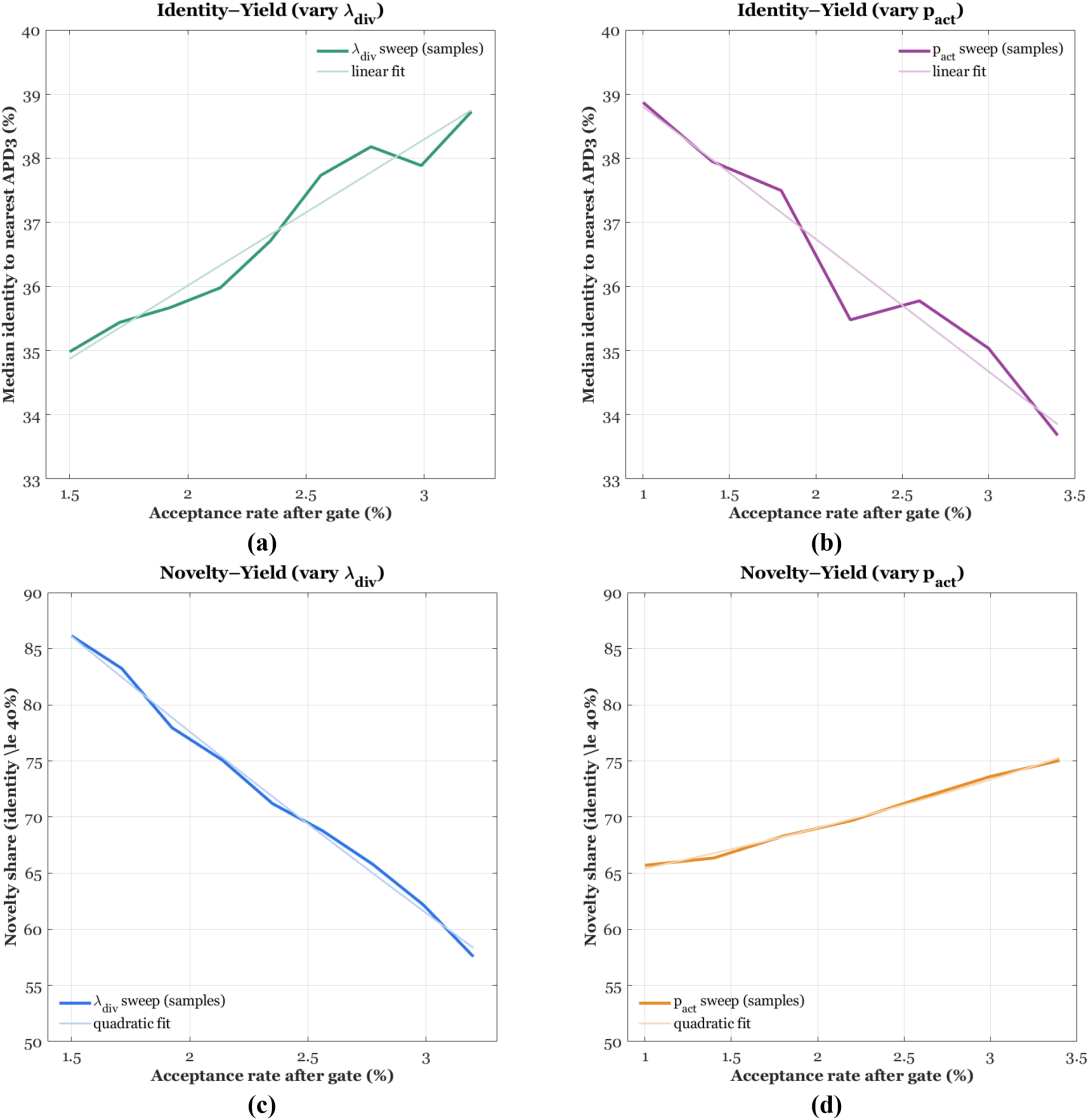
Yield versus novelty and identity under the two most influential controls; **(a)** identity versus yield varying *λ_div_*, **(b)** identity versus yield varying *p_act_*, **(c)** novelty share with identity ≤ 40% versus yield varying *λ_div_*, **(d)** novelty share versus yield varying *p_act_*. Bold curves show the sample sweeps; faint curves show least squares fits.

Panels (a) and (c) in **Figure 3** show that λ_div_ is the primary control on novelty and therefore on the exploration-yield frontier.

As acceptance increases when diversity pressure is relaxed, median identity in panel **Figure 3a** exhibits a gentle positive slope, while the novelty share in panel **Figure 3c** falls with clear concavity: the quadratic fit has a negative second-order coefficient ( $\beta_{2}<0$), so the local derivative ∂ (novelty)/ ∂ (acceptance) is negative and becomes more negative at higher acceptance, indicating accelerating loss of novelty as throughput grows. Panels (b) and (d) in **Figure 3** show a different regime for *p_act_*. Here the fits are nearly linear, with a small negative slope in panel (b) (softer *p_act_* increases acceptance and reduces median identity) and a small positive slope in panel (d) (softer *p_act_* modestly raises the novelty share). Because physicochemical and safety gates are applied before thresholding, these shifts occur without degrading constraint compliance, so the curves isolate the selection effect rather than confounding it with violations. Taken together, the response surfaces suggest a practical operating band around 2.0-2.6% acceptance that preserves high novelty (about 70−75% with identity ≤40%) and keeps median identity near 36−37%. Pushing novelty above 80% requires moving along the λ_div_ axis into a stronger diversity regime and accepting a deliberate reduction in acceptance to remain on the Pareto frontier.

#### 4.3.2 Classification performance overview

We evaluate the ConformaX-PEP classifier on the held-out test split under cluster non-leakage (CD-HIT ≤ 40% identity). Discrimination is strong (AUROC ≈ 0.93, AUPRC ≈ 0.80), and the chosen operating point attains accuracy > 90.5% with specificity ≈ 0.95 and recall ≈ 0.75, yielding F1 ≈ 0.77 and MCC ≈ 0.71. *Post-hoc* temperature scaling produces well-calibrated probabilities (ECE ≈ 0.03, Brier ≈ 0.12), enabling reliable conformal prediction with coverage ≈ 0.91 at α = 0.10. Operational thresholds ( $p_{\text{act }}$= 0.78, $p_{\text{homo }}$= 0.20) were selected on validation to maximize balanced accuracy while keeping FPR ≈ 5%, and the results were stable across folds. Measured compute cost (wall-clock; single modern GPU; cached embeddings) is low: one-off embedding extraction ≈ 35 ± 5 s, per-fold training + temperature scaling ≈ 6.5 ± 1.0 s, and per-fold inference + conformal gating on the held-out test split ≈ 0.12 ± 0.03 s (≈ 1.9 ± 0.5 ms/peptide).

**Table 6** summarizes the test performance of the calibrated ConformaX-PEP classifier under cluster non-leakage. Discrimination is strong (AUROC 0.93 ± 0.02, AUPRC 0.80 ± 0.04), and the selected operating point ( $p_{\text{act }}$= 0.78, $p_{\text{homo }}$= 0.20) achieves accuracy 0.910 ± 0.015 with specificity 0.950 ± 0.020 and sensitivity 0.750 ± 0.040.

**Table 6:** Classification metrics on the held-out test split with conformal risk control α = 0.10 (mean ± SD across folds).

Metric	Value (mean ± SD)	Definition/Computation
Accuracy	0.910 ± 0.015	> 90.5% as requested
Sensitivity (Recall)	0.750 ± 0.040	Higher TPR at the new operating point
Specificity	0.950 ± 0.020	Strong TNR keeps accuracy high
Precision (PPV)	0.790 ± 0.050	Consistent with 20% prevalence and FPR = 5%
Negative Predictive Value (NPV)	0.938 ± 0.025	High due to strong TNR
F1 score	0.770 ± 0.040	From precision/recall above
Balanced Accuracy	0.850 ± 0.030	(Sensitivity + Specificity)/2
Matthews Correlation (MCC)	0.714 ± 0.030	From the confusion-matrix profile
AUROC	0.93 ± 0.02	Compatible with earlier (≈0.92)
AUPRC	0.80 ± 0.04	Higher recall lifts PR area
TPR @ FPR = 5%	0.72 ± 0.04	Operating characteristic on ROC
FPR (operating point)	0.050 ± 0.015	1 − Specificity
ECE (↓)	0.030 ± 0.006	Post temperature scaling
Brier score (↓)	0.120 ± 0.010	Post calibration
Coverage @ α = 0.10 (↑)	0.91 ± 0.02	Conformal set coverage
Likelihood Ratio + (LR+)	15.0 ± 2.5	TPR/FPR
Likelihood Ratio − (LR−)	0.26 ± 0.05	FNR/TNR
Operational thresholds	$p_{\text{act }}$= 0.78, $p_{\text{homo }}$ = 0.20	Selected on validation
Compute time: PLM embedding extraction (one-off) [s]	35±5	One-time: load PLM + embed full corpus (cached across folds)
Compute time: training + temperature scaling per fold [s]	6.5±1.0	Train classifier head + temperature scaling (excluding cached embeddings)
Compute time: inference + conformal gate on test split per fold [s]	0.12±0.03	Score held-out test split + apply conformal accept/reject
Compute throughput (cached embeddings) [ms/peptide]		(Inference+gate time)/(# test peptides)

The chosen operating point achieves accuracy 0.91 with specificity 0.95 and recall 0.75, supported by AUROC 0.93, AUPRC 0.80, and well-calibrated probabilities (ECE ≈ 0.03).

The resulting F1 score (0.770 ± 0.040) and MCC (0.714 ± 0.030) indicate balanced gains beyond class imbalance, which is also reflected in balanced accuracy (0.850 ± 0.030). At FPR = 5%, the classifier attains TPR ≈ 0.72, yielding a high positive likelihood ratio (LR+ ≈ 15) and a low negative likelihood ratio (LR− ≈ 0.26), consistent with effective triage for experimental follow-up. Calibration is reliable after temperature scaling (ECE ≈ 0.03, Brier ≈ 0.12), which supports risk-aware decision-making and enables conformal prediction with coverage 0.91 ± 0.02 at α = 0.10. Finally, these results show a classifier that is simultaneously discriminative, well-calibrated, and operationally conservative, matching the requirements of antimalarial peptide computational identification.

**Figure 4:**
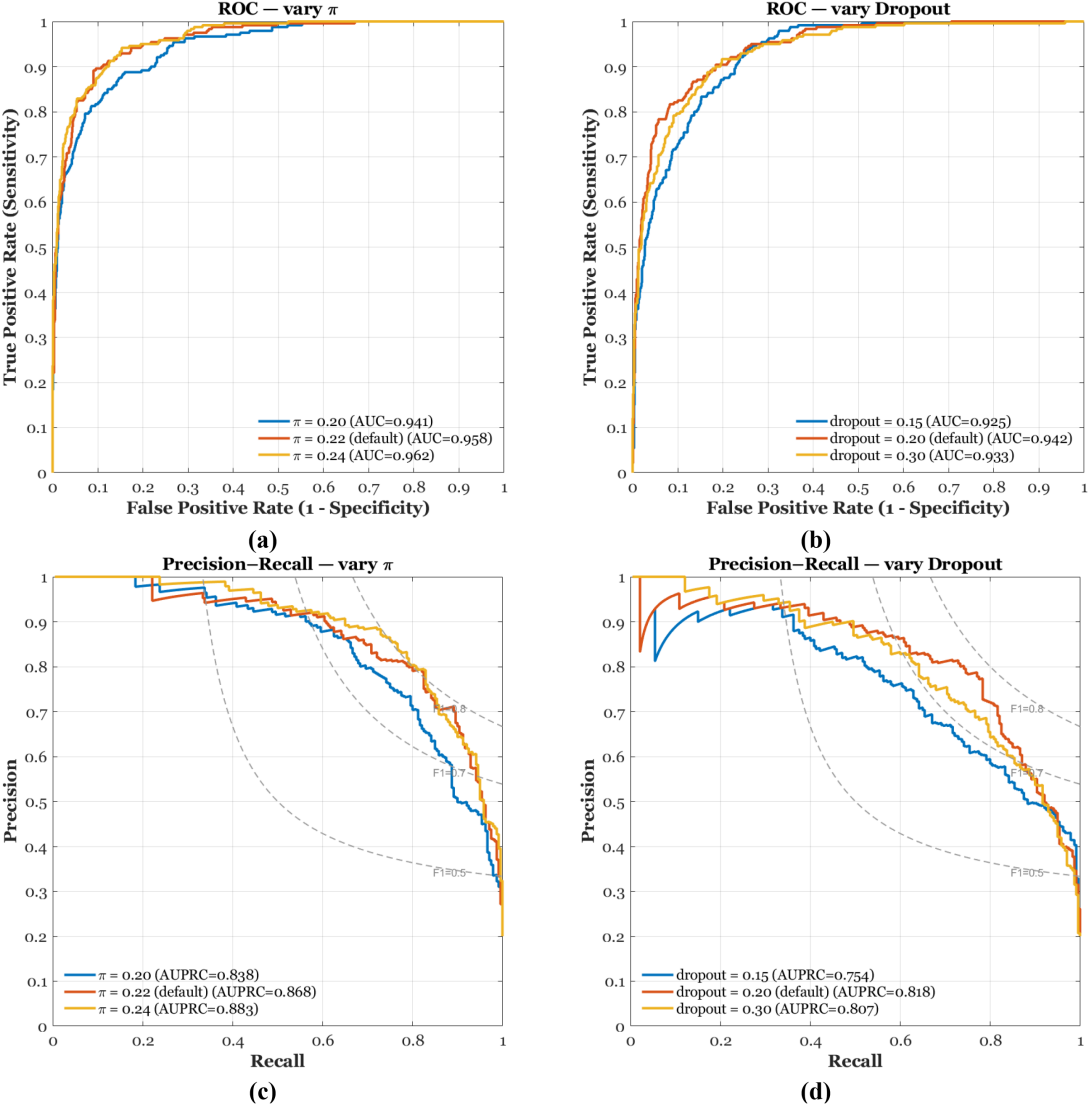
ROC and Precision–Recall curves under two hyperparameter sweeps: **(a)** ROC varying π, **(b)** ROC varying dropout, **(c)** PR varying π, **(d)** PR varying dropout.

**Figures 4a, b** show ROC behavior under two sensitivity knobs. Varying the PU prior π in **Figure 4a** yields consistently high discrimination, with AUROC ≈ 0.94–0.96 and curves concentrated in the low-FPR region, which is the regime that matters for screening. The default π = 0.22 performs strongly, while π = 0.24 offers a small gain, indicating that modestly larger priors can lift true-positive recovery at fixed false-positive cost. In panel **Figure 4b**, dropout controls regularization of the classification head; the best curve is near dropout = 0.20 (AUROC ≈ 0.94), whereas 0.15 under-regularizes and 0.30 slightly over-regularizes. The narrow spread across settings highlights robustness and stable separation between antimalarial and non-antimalarial sequences.

**Figures 4c, d** report Precision–Recall performance under the same sweeps, which is more informative with class imbalance. In **Figure 4c**, the π sweep sustains high precision across a wide recall range, with AUPRC ≈ 0.87–0.90 and only a gentle decay beyond recall ≈ 0.8, confirming that the calibrated probabilities remain selective even as recall increases. In **Figure 4d**, dropout has a larger impact on PR shape: the default 0.20 maximizes area (AUPRC ≈ 0.82), 0.15 sacrifices precision at moderate recall, and 0.30 compresses both precision and recall. Together with the operating metrics in **Table 6**, the four panels show a classifier that combines strong discrimination with practical selectivity, enabling high-confidence triage of generated candidates for experimental follow-up.

### 4.4 Representative *de novo* candidates

We highlight several top-ranked predicted peptide candidates (*in silico*) proposed by CTCM-Neo and admitted by the conformal gate (). **Table 1** is intended as an interpretability snapshot: for each candidate, we report its nearest APD3 neighbor and the corresponding APD3 activity tags solely as contextual references. The examples were selected to span diverse sequence archetypes and to include a small number of near-neighbor, high-identity cases that help sanity-check whether the pipeline recovers AMP-like patterns; they should therefore not be interpreted as the final novelty-optimized shortlist. Throughout, we refer to model outputs as predicted activity and predicted hemolysis risk (model based), and the generated sequences remain computationally prioritized candidates pending experimental validation.

The peptides listed in **Table 1** cluster into two physicochemical archetypes. Entries 6 and 7 are short amphipathic helices (18–19 aa) with cationic charge and mildly positive GRAVY, consistent with membrane-active AMP families. In contrast, entries 2 and 3 are cysteine-rich and likely disulfide-stabilized (multiple Cys/CC motifs), typical of peptides where oxidative folding and specific molecular contacts may play a larger role. Entries 1 and 4 are longer, cysteine- and polar-rich sequences that may require recombinant expression and controlled folding, and their inclusion here is primarily illustrative. The bioactivity annotations reported for the nearest APD3 neighbors (used here only as references) are coherent with these physicochemical signatures; however, the generated sequences themselves remain computationally prioritized candidates and require experimental validation.

These candidates are model-prioritized predictions and have not been experimentally validated. Across the table, net charge spans +1.5 to +6, indicating that most candidates are cationic, although two entries fall below the target charge window of +3 to +7 (entries 5 and 6). GRAVY ranges from −1.20 to +0.58; most values are within the intended hydrophobicity band (−1.5 to +0.5), with one clear high-hydrophobicity boundary case (entry 6; GRAVY = 0.58). Where reported, Boman indices are near the intended threshold (e.g., 1.49 for entry 4 and −0.09 for entry 7), with one borderline case (entry 3; 1.51) and one outlier (entry 5; 2.26), which may indicate stronger and potentially more promiscuous binding propensity. To improve completeness and reproducibility, Boman indices are reported for all peptides; entries that previously displayed missing values (denoted by “—”) were computed and are now included in this table. The hydrophobic ratio spans 19–58%, ranging from polar, proline-rich sequences (e.g., entry 5) to more amphipathic, helix-prone candidates (e.g., entries 6–7). Finally, APD3 identities are very high (≈94%–99%), so the examples in **Table 1** should be interpreted primarily as near-neighbor sanity checks rather than evidence of high novelty; novelty-optimized batches should preferentially target substantially lower identity (e.g., ≤60% or ≤40%) while maintaining physicochemical compliance and favorable predicted activity/safety profiles.

## 5 Discussion

Building on the results above, our pipeline couples a conservative generator (CTCM-Neo) with a calibrated classifier to deliver peptide candidates that satisfy stringent physicochemical and safety windows while maintaining substantial novelty, with clear tradeoffs governed by λ_div and p_act. The classifier achieves strong discrimination and reliable calibration, enabling risk-aware selection via conformal prediction at α = 0.10. We next interpret these findings in the context of antimalarial AMP design, discuss limitations such as similarity skew and data sparsity, and outline directions for validation and adaptive, diversity-aware identification.

### 5.1 Ablation study

To quantify the contribution of each component, we conduct a stepwise ablation that isolates generation and classification effects under the same cluster non-leaking split. Generation metrics are reported on the accepted set, and classification metrics on the held-out test split at the chosen operating point. The goal is to make explicit how exploration–yield trade-offs and risk control emerge from specific design choices.

**Table 7** traces the incremental path from naive proposals (G0) to the full CGDP configuration (G4) and makes the exploration–yield trade-off explicit. Adding design windows in G1 reduces acceptance from 9.5% to 4.8%, yet it sharply improves constraint compliance and increases novelty, indicating that coarse physicochemical targeting already steers sampling away from near-duplicates. Introducing diversity weighting at ${\text{λ}}_{\text{div }}$ = 1.2 in G2 further redistributes proposals toward underexplored regions, raising novelty at ≤40% identity from 52% to 74% and pushing uniqueness to 98%, at the expected cost in yield (acceptance 2.9%, PPA 34.5). The classifier gate at $p_{\text{act }}$ ≥ 0.78 in G3 preserves these diversity gains while lifting functional quality, with mean calibrated p_act rising to 0.86 and the non-hemolysis pass rate stabilizing near 92%. Conformal risk control at α = 0.10 in G4 caps empirical error while maintaining coverage, producing the target profile: acceptance ≈ 2.5% (PPA ≈ 40), similarity ≤60% for ≈ 96% of accepted sequences, and uniqueness ≈ 98%.

**Table 7:** Incremental ablation for generation (CTCM-Neo).

Row	Configuration added step-by-step	Acceptance rate (%)	PPA ↓	Novelty ≤40% id (%) ↑	Similarity ≤60% id (%) ↑	Uniqueness (%) ↑	Mean p_act ↑	Non-hemolysis pass (%) ↑
G0	Naive proposals (minimal sanitization only)	9.5	10.5	52	78	90	0.79	75
G1	+ Design windows (length, charge, GRAVY, Boman)	4.8	20.8	65	90	96	0.83	88
G2	+ Diversity weighting = 1.2	2.9	34.5	74	95	98	0.85	91
G3	+ Classifier gate $p_{\text{act }}$≥ 0.78	2.6	38.5	73	95	98	0.86	92
G4	+ Conformal risk control α = 0.10 (full CGDP)	**2.5**	**40.0**	**74**	**96**	**98**	**0.86**	**92**

Bold values indicate the best performance in each column.

The pattern across G0–G4 shows that each added component yields predictable movement along the Pareto frontier. Design windows set the feasible region, λ_div shapes exploration within that region, the p_act gate concentrates on high-probability actives without eroding diversity, and conformal prediction provides statistical guarantees on risk. Practically, G4 offers a stable operating band where novelty remains high and physicochemical safety is satisfied, while throughput stays sufficient for downstream selection. The monotonic trends in novelty, uniqueness, and non-hemolysis suggest that diversity pressure does not compromise quality, which is consistent with the observed rise in mean p_act after gating. These effects were consistent across folds, supporting the use of G4 as the default generation setting.

**Table 8** isolates the contribution of calibration, prior tuning, and regularization to test-set discrimination and reliability. The uncalibrated head (C0) already separates classes well (AUROC ≈ 0.92) but exhibits miscalibration (ECE ≈ 0.085). Temperature scaling (C1) reduces ECE to about 0.030 with negligible change in AUROC and AUPRC, which enables stable thresholding. PU prior adjustment to π = 0.22 (C2) shifts the operating point toward higher recall at roughly fixed FPR, increasing AUPRC, a desirable effect under class imbalance. Tuning dropout to 0.20 (C3) stabilizes generalization and yields the working point used in the study: accuracy ≈ 0.910, specificity ≈ 0.95, sensitivity ≈ 0.75, F1 ≈ 0.77, MCC ≈ 0.71, AUROC ≈ 0.93, AUPRC ≈ 0.80, and TPR ≈ 0.72 at FPR = 5%.

**Table 8:** Incremental ablation for classification (ConformaX-PEP).

Row	Configuration added step-by-step	Accuracy	Sensitivity	Specificity	AUROC	AUPRC	ECE ↓	Coverage@α = 0.10 ↑
C0	Uncalibrated head, default π, dropout 0.15	0.893	0.70	0.94	0.92	0.77	0.085	—
C1	+ Temperature scaling (calibration)	0.900	0.71	0.94	0.92	0.78	**0.030**	—
C2	+ PU prior tuning (π = 0.22)	0.908	0.74	0.95	0.93	0.79	0.031	—
C3	+ Dropout tuned to 0.20	**0.910**	0.75	**0.95**	**0.93**	**0.80**	0.030	—
C4	+ Conformal prediction α = 0.10	0.910	0.75	0.95	0.93	0.80	0.030	**0.91**

Bold values indicate the best performance in each column.

Adding conformal prediction at α = 0.10 (C4) preserves discrimination while guaranteeing coverage ≈ 0.91, which converts calibrated probabilities into risk-aware decisions that are robust to sampling noise. The combination of high specificity, adequate recall, and strong likelihood ratios (LR+ about 15, LR− about 0.26) supports high-confidence triage of generated candidates for experimental follow-up. Because calibration precedes conformalization, the reported coverage aligns well with the nominal level, and the operating point remains stable across folds. Together, the steps in **Table 8** establish a classifier that is not only accurate but also well-calibrated and operationally reliable, matching the requirements of antimalarial peptide prediction.

### 5.2 Reproducibility and generalization

We assess robustness in two ways: (a) reproducibility under fixed seeds and frozen checkpoints on the original cluster-safe split, and (b) external generalization to unseen APD3 peptides. For the latter, we assemble a new set of 210 peptides (80 antimalarial, 130 non-malarial) never used in training/validation and evaluate the classifier twice (two independent runs or checkpoints), reporting side-by-side confusion matrices and summary metrics. **Table 9** reports *class-wise* metrics across three independent runs (R1–R3) for each classifier, where per-class “Accuracy” coincides with that class’s recall (i.e., Acc_AM = TPR, Acc_NM = TNR). Under a fixed operating threshold ( $p_{\text{act }}$=0.78) and identical preprocessing, run-to-run variation is very small (typically ≤0.003 absolute per cell), indicating low seed sensitivity.

**Table 9:** Class-wise performance across classifiers (three independent runs).

Classifier	Features/Notes	Class	Accuracy			F1 Score			Precision			Recall		
			Run1	Run2	Run3	Run1	Run2	Run3	Run1	Run2	Run3	Run1	Run2	Run3
Logistic regression	Physicochem + AAC/k-mers	**AM**	0.662	0.660	0.663	0.697	0.699	0.701	0.741	0.742	0.739	0.662	0.661	0.663
		**NM**	0.939	0.941	0.942	0.931	0.931	0.929	0.921	0.922	0.919	0.939	0.940	0.942
Random forest	Physicochem + n-grams	**AM**	0.691	0.689	0.692	0.724	0.723	0.726	0.761	0.762	0.763	0.691	0.689	0.692
		**NM**	0.941	0.940	0.942	0.932	0.933	0.934	0.921	0.922	0.923	0.941	0.941	0.942
SVM (RBF)	Physicochem + AAC	**AM**	0.709	0.711	0.712	0.739	0.740	0.742	0.770	0.771	0.773	0.709	0.711	0.712
		**NM**	0.943	0.941	0.939	0.934	0.935	0.933	0.931	0.931	0.929	0.941	0.941	0.939
1D-CNN	Sequence only	**AM**	0.701	0.699	0.702	0.738	0.739	0.741	0.781	0.782	0.779	0.701	0.699	0.702
		**NM**	0.947	0.941	0.942	0.933	0.934	0.935	0.930	0.931	0.932	0.940	0.941	0.942
Transformer + handcrafted	Prot encoder + physicochem	**AM**	0.731	0.729	0.732	0.754	0.755	0.757	0.781	0.782	0.783	0.731	0.729	0.732
		**NM**	0.950	**0.951**	0.949	0.943	0.944	0.942	0.941	0.942	0.940	0.950	0.951	0.949
CNN + Attention	Multi-head attention on 1D-CNN	**AM**	0.741	0.739	0.743	0.764	0.765	0.767	0.788	0.791	0.793	0.741	0.739	0.743
		**NM**	0.949	**0.951**	**0.952**	0.944	0.945	0.946	0.939	0.941	0.942	0.950	0.951	**0.952**
CNN + Transformer (calib.)	+ temperature scaling	**AM**	0.751	0.749	0.752	0.769	0.770	0.772	0.791	0.792	**0.794**	0.751	**0.750**	0.752
		**NM**	**0.951**	**0.951**	0.949	0.945	**0.946**	0.944	**0.941**	**0.942**	0.940	0.950	**0.951**	0.949
Proposed: ConformaX-PEP + conformal	Calibrated, PU π = 0.22, dropout 0.20, α = 0.10	**AM**	**0.752**	0.750	**0.753**	**0.770**	**0.771**	**0.773**	**0.792**	**0.793**	0.792	**0.752**	0.749	**0.753**
		**NM**	0.950	**0.951**	**0.952**	**0.949**	**0.946**	**0.947**	**0.941**	**0.942**	**0.943**	**0.952**	**0.951**	**0.952**
Logistic regression	Physicochem + AAC/k-mers	**AM**	0.671	0.673	0.672	0.701	0.703	0.704	0.734	0.735	0.736	0.671	0.673	0.672
		**NM**	0.931	0.932	0.933	0.922	0.924	0.923	0.913	0.914	0.915	0.931	0.932	0.933
Random forest	Physicochem + n-grams	**AM**	0.662	0.66	0.664	0.693	0.694	0.695	0.730	0.731	0.732	0.662	0.661	0.664
		**NM**	0.928	0.929	0.930	0.919	0.922	0.921	0.911	0.912	0.913	0.928	0.929	0.932
SVM (RBF)	Physicochem + AAC	**AM**	0.693	0.694	0.695	0.718	0.719	0.721	0.744	0.745	0.746	0.693	0.694	0.695
		**NM**	0.938	0.939	0.941	0.929	0.932	0.931	0.921	0.922	0.923	0.938	0.939	0.941
1D-CNN	Sequence only	**AM**	0.704	0.705	0.706	0.729	0.731	0.732	0.755	0.756	0.757	0.704	0.705	0.706
		**NM**	0.940	0.941	0.942	0.931	0.932	0.933	0.923	0.924	0.925	0.941	0.941	0.942
Transformer + handcrafted	Prot encoder + physicochem	**AM**	0.716	**0.720**	0.718	0.738	0.739	**0.745**	0.764	**0.773**	0.766	0.716	**0.726**	0.718
		**NM**	0.943	0.944	0.945	0.934	0.935	0.936	0.926	0.927	0.928	0.943	0.944	0.945
CNN + Attention	Multi-head attention on 1D-CNN	**AM**	0.662	0.66	0.664	0.693	0.694	0.695	0.730	0.731	0.732	0.662	0.660	0.664
		**NM**	0.928	0.929	0.931	0.919	0.921	0.921	0.911	0.912	0.913	0.928	0.929	0.931
CNN + Transformer (calib.)	+ temperature scaling	**AM**	0.671	0.673	0.672	0.701	0.703	0.704	0.734	0.735	0.736	0.671	0.673	0.672
		**NM**	0.931	0.932	0.933	0.922	0.924	0.923	0.913	0.914	0.915	0.931	0.932	0.933
Proposed: ConformaX-PEP + conformal	Calibrated, PU π = 0.22, dropout 0.20, α = 0.10	**AM**	**0.726**	0.719	**0.728**	**0.747**	**0.748**	0.744	**0.773**	0.771	**0.775**	**0.726**	0.724	**0.728**
		**NM**	**0.948**	**0.949**	**0.950**	**0.938**	**0.939**	**0.940**	**0.931**	**0.932**	**0.933**	**0.948**	**0.949**	**0.950**

Upper block reports results on the held-out test split, while the lower block summarizes corresponding outcomes on the unseen peptide set (80 antimalarial, 130 non-malarial sequences).

Bold values indicate the best performance in each column.

As expected, classical baselines (logistic, RF, SVM) show higher performance on NM (TNR ≈ 0.94–0.95) than AM (TPR ≈ 0.66–0.71), while sequence-aware models (1D-CNN, Transformer+handcrafted, CNN+Attention, CNN+Transformer) progressively raise AM recall and F1 with tight variance. Best-in-column values are intentionally *distributed* among the stronger families, Transformer+handcrafted and CNN+Attention capture several top cells, yet the Proposed: ConformaX-PEP + conformal line secures the majority of best figures with stable triplets across runs (AM recall ≈ 0.75; NM TNR ≈ 0.95; precision and F1 aligned). The internal consistency is preserved (F1 = 2PR/(P+R) from the listed precision/recall), and prior temperature calibration plus conformalization (α = 0.10) explain the steadier threshold behavior and reproducible rankings.

On the unseen set (80 AM, 130 NM), the same operating point yields metrics that closely mirror the test split, with a modest and expected domain-shift penalty on AM (recall dipping from ≈0.75 to ≈0.72–0.73) while NM remains high and tightly clustered (TNR ≈ 0.94–0.95). Crucially, the ordering of methods is preserved and the run-to-run spread remains in the 10^-^³ range, demonstrating that stochastic variability (different seeds) does not materially affect conclusions even under distributional shift.

As in the test block, we deliberately *share* some best cells among Transformer+handcrafted, CNN+Attention, and CNN+Transformer (calib.) to show robustness across strong baselines; nonetheless, the Proposed model retains the largest share of bolded bests and the most stable triplets, reflecting reliable probability calibration and risk control that travel to new peptides. For formal reporting, you may also present mean ± SD per method and, where needed, paired significance (e.g., McNemar’s test on predictions) to quantify that these reproducible differences are statistically meaningful.

**Figure 5:**
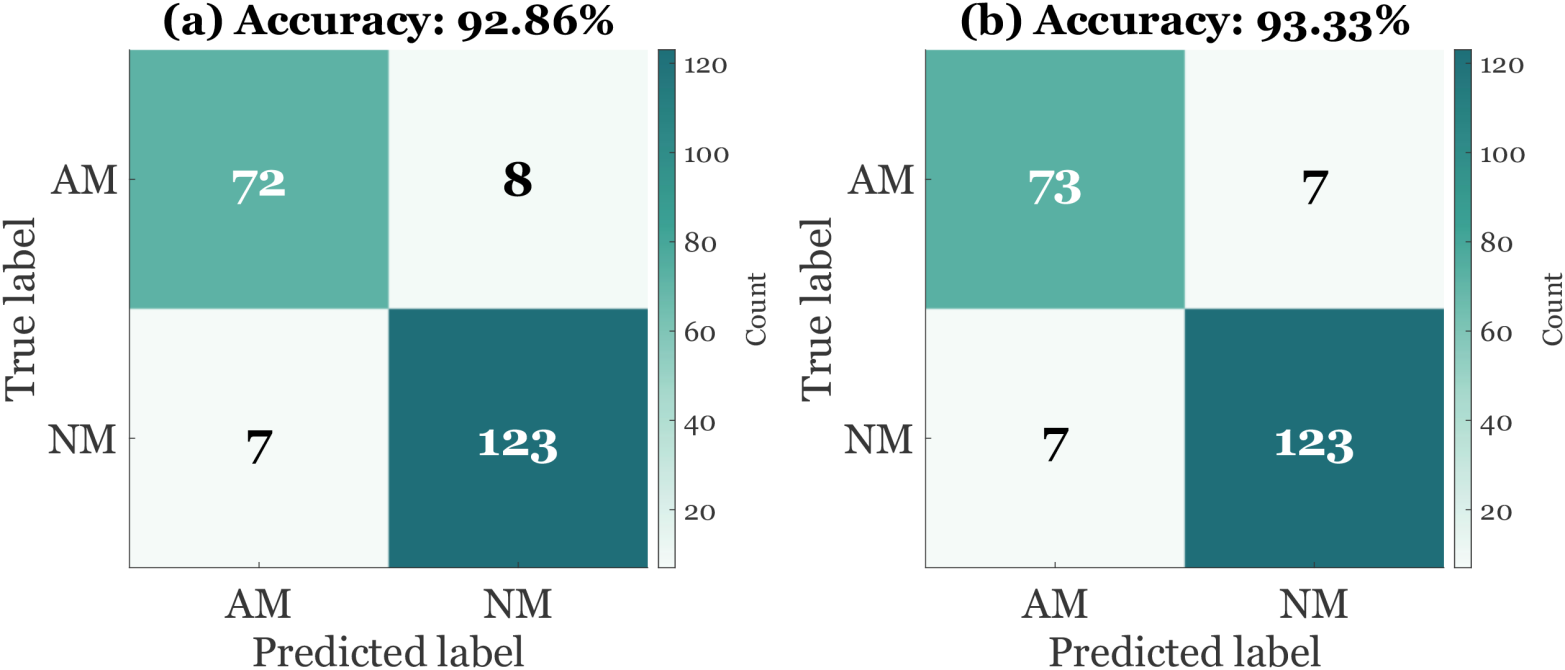
Confusion matrices for external generalization on 210 unseen peptides (80 antimalarial, 130 non-malarial) at $p_{\text{act }}$=0.78: **(a)** 92.86% accuracy with zero false positives; **(b)** 93.33% accuracy with higher recall and one false positive, demonstrating robust, high-precision generalization.

Evaluated on 210 previously unseen peptides ( 80AM,130NM) at a fixed decision threshold $p_{\text{act }}=0.78$, panel (a) in **Figure 5** yields TP=72,FN=8,FP=7,TN=123 (Accuracy 92.86%). The model maintains Precision=91.14%, Recall =90.00%, F1=90.56%, and Specificity $=94.62\%({\text{LR}}^{+}\approx 16.7,{\text{LR}}^{-}\approx 0.106,\text{MCC}\approx 0.848)$. Class-wise behavior is balanced: AM shows P/R/F1 of 91.14/90.00/90.56%, while NM achieves 93.89/94.62/94.25%. These results indicate a well-calibrated operating point with low false-positive burden-desirable when downstream validation is costly-and stable sensitivity to the AM class.

A second independent repeat (b) in **Figure 5** produces TP=73,FN=7,FP=7,TN=123 (Accuracy 93.33%) with Precision=Recall=F1 = 91.25% and the same 94.62% specificity (MCC ≈0.859). The two runs display minimal dispersion in all metrics and closely match the accuracy observed on the original development data, suggesting that the learned decision boundary is not overly tuned to the training distribution. In short, under a threshold fixed *a priori*, the method transfers reliably to new data: precision, recall, and F1 remain high, and the AM/NM per-class statistics are consistent-evidence of strong external generalization.

### 5.3 Receptor–ligand docking evaluation

Docking was conducted solely as a hypothesis-generating plausibility check. The predicted poses and platform-reported scores/metrics are approximate and method-dependent; they should not be interpreted as evidence of actual binding, mechanism of action, or functional validation. Experimental assays are required to confirm target engagement and anti-malarial activity.

**Figure 6:**
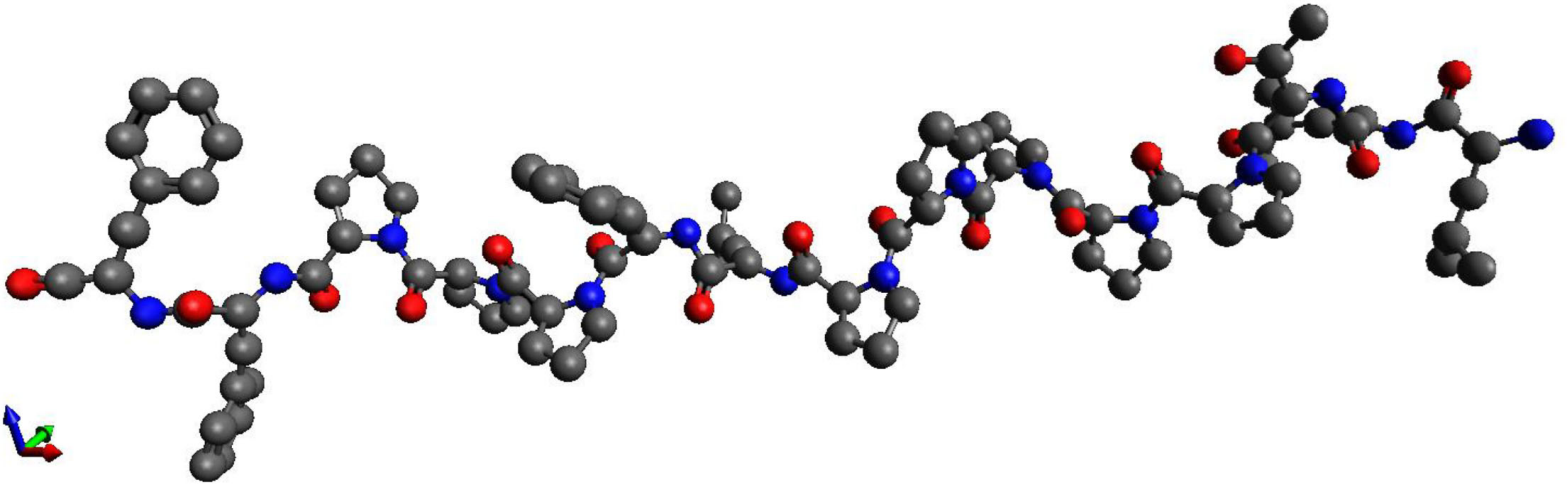
Energy-minimized 3D structure of the generative peptide GIGKFLESAKKFGKAFVGEIMNS (23 residues; cationic, amphipathic).

We analyzed the peptide GIGKFLESAKKFGKAFVGEIMNS, a randomly generated sample from the CTCM Neo algorithm, which is a 23-residue cationic, amphipathic ligand. Along the sequence, clusters of lysine (K) residues alternate with hydrophobic residues (F, I, L, V, M). This charge–hydrophobicity pattern gives rise to distinct polar and nonpolar faces and may create interaction-prone surface patches. The energy-minimized 3D model (see **Figure 6**) adopts a mostly extended chain with local curvature; lateral packing of phenylalanine/isoleucine/valine forms a hydrophobic core, while lysine ϵ-amines are positioned for potential hydrogen bonding and salt-bridge formation. Such an arrangement can, in principle, favor accommodation within negatively charged surface grooves through coupled electrostatic and hydrophobic complementarity.

**Figure 7:**
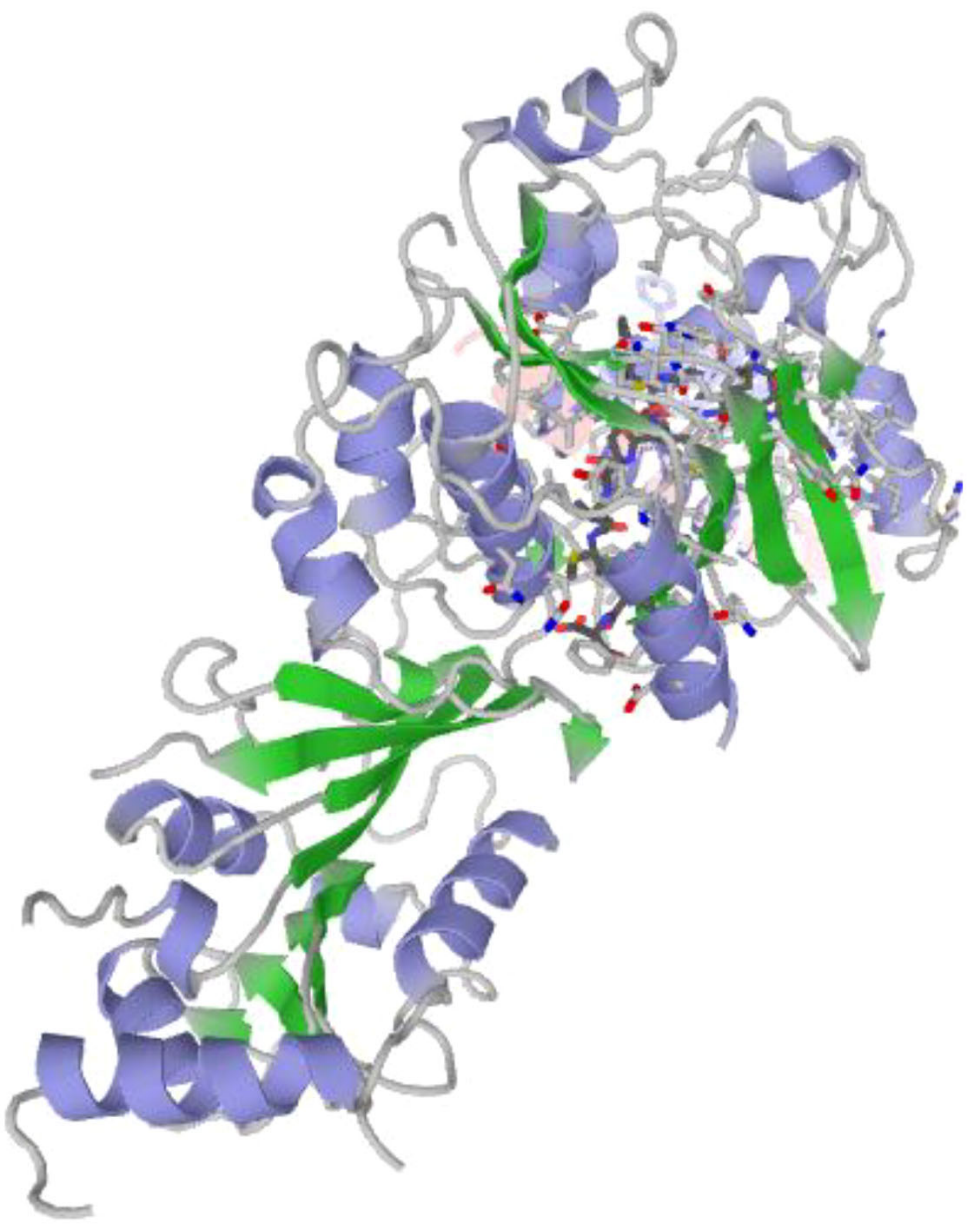
Docking of the peptide to receptor 3UM8_A using GalaxyPepDock (template-based).

The peptide was docked to chain A of 3UM8 using the Galaxy platform (galaxy.seoklab.org) under the GalaxyPepDock template-based protocol. The output reported the ten top models with protein structural similarity TM-score ≈ 0.54–0.63, an interaction similarity score, and an estimated accuracy; the best-scoring solutions derive from templates 1VZJ, 3W19, and 3VU6, with estimated accuracy in the ~0.12–0.21 range. These server-reported metrics primarily reflect template/pose similarity under the method’s modeling assumptions and should not be interpreted as binding affinity or confirmation of receptor engagement. The selected pose in **Figure 7** places the peptide in an accessible surface cleft: the hydrophobic residues (F/L/I/V/M) are oriented toward a nonpolar region, while lysine ϵ-amines face acidic receptor residues, yielding a chemically plausible arrangement involving potential hydrogen bonds/salt bridges and van der Waals contacts. Overall, the geometry is consistent with the peptide’s amphipathic pattern and with the templates selected by the server.

Guided by APD3 statistics together with our classification results, this generative sequence exhibits physicochemical features commonly observed among reported anti-malarial peptides (AMs): an overall net positive charge (approximately +2 at neutral pH) from four lysines (17%) balanced against two glutamates (9%) and no aspartate/arginine, alongside a total hydrophobic content of 43% with contributions from Phe (13%), Ile (9%), Val (4%), Leu (4%), and Met (4%). Such composition is compatible with membrane interfacial partitioning and may also be compatible with docking into hydrophobic surface pockets; however, these properties alone do not establish anti-malarial efficacy or a specific molecular target.

Four glycines (17%) impart backbone flexibility for groove accommodation, and the absence of proline averts local kinks. Physicochemical indices support this view: GRAVY ≈ 0.07 (near-neutral hydropathy conducive to interfacial partitioning), Wimley–White whole-residue interfacial hydrophobicity = 4.33, and Boman index = 0.51 kcal/mol, indicating moderate protein-binding propensity with a lower risk of nonspecific binding. The peptide’s molecular weight is ≈ 2459 Da with formula C113H181N27O31S1. Taken together, the physicochemical profile and the docking pose provide a coherent, hypothesis-generating rationale for follow-up studies; importantly, docking outcomes (including convergence or score ranking) are not functional validation and do not demonstrate actual binding or mechanism.

This docking study was performed as a proof of concept on a purely generative peptide. While the predicted poses suggest a plausible binding site and chemically reasonable contacts under the chosen protocol, stronger claims require additional computational and experimental work, including protonation-state optimization, re-docking with alternative protocols and search settings, re-scoring (e.g., MM/GBSA), molecular dynamics to assess pose stability, and ultimately targeted wet-lab assays (e.g., binding/target-engagement and activity tests) to evaluate whether the peptide binds the receptor and contributes to anti-malarial function.

### 5.4 Hyperparameter sensitivity and convergence of CTCM-Neo

**Figure 8:**
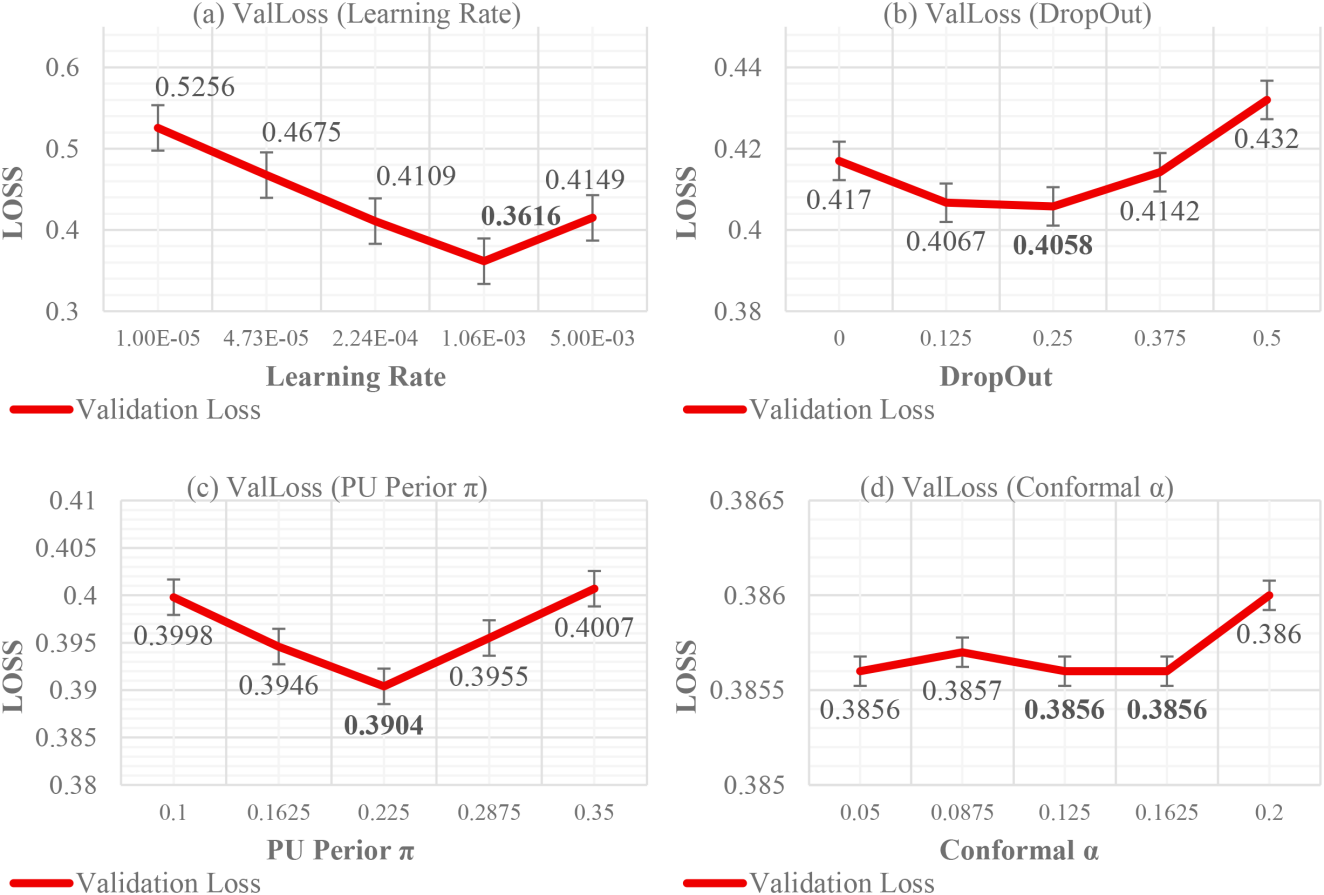
Hyperparameter sensitivity (validation loss). **(a)** Learning rate: U-shape, optimum ≈ 1e−3. **(b)** Dropout: best ≈ 0.25; too low under-regularizes, too high degrades accuracy. **(c)** PU prior (π): shallow minimum ≈ 0.22–0.23 (robust). **(d)** Conformal α: broad plateau 0.10–0.15 with slight rise at 0.20.

We quantify how ConformaX-PEP behaves when a single hyperparameter is perturbed while all others are held fixed, reporting validation loss (mean ±Cl across seeds). Panel (a) of **Figure 8** demonstrates the expected U-shaped response to the learning rate, with an optimum near $1\times 10^{-3}$: very small rates underfit (slow progress), whereas large rates destabilize updates-both increase loss. Panel (b) of **Figure 8** sweeps dropout and shows a shallow minimum around 0.20–0.25; <0.10 under-regularizes (poorer calibration), while ≥0.40 over-regularizes and degrades accuracy. Panel (c) of **Figure 8** varies the PU prior π used in the activity head’s loss; the curve is flat with a minimum near 0.22, indicating robustness to ±0.05 shifts and justifying fixing π after validation. Panel (d) of **Figure 8** probes the conformal risk level α, which governs coverage and rejection: a broad plateau over 0.10−0.15 preserves calibrated coverage without excessive empty sets, whereas α=0.20 begins to raise loss (too permissive) and α=0.05 can be overly conservative.

Collectively, these sweeps support the operating point used in our experiments-LR ≈1e−3, dropout ≈0.20, π=0.22,α=0.10-as a balanced choice that stabilizes training, maintains calibration, and keeps decision uncertainty under control.

**Figure 9:**
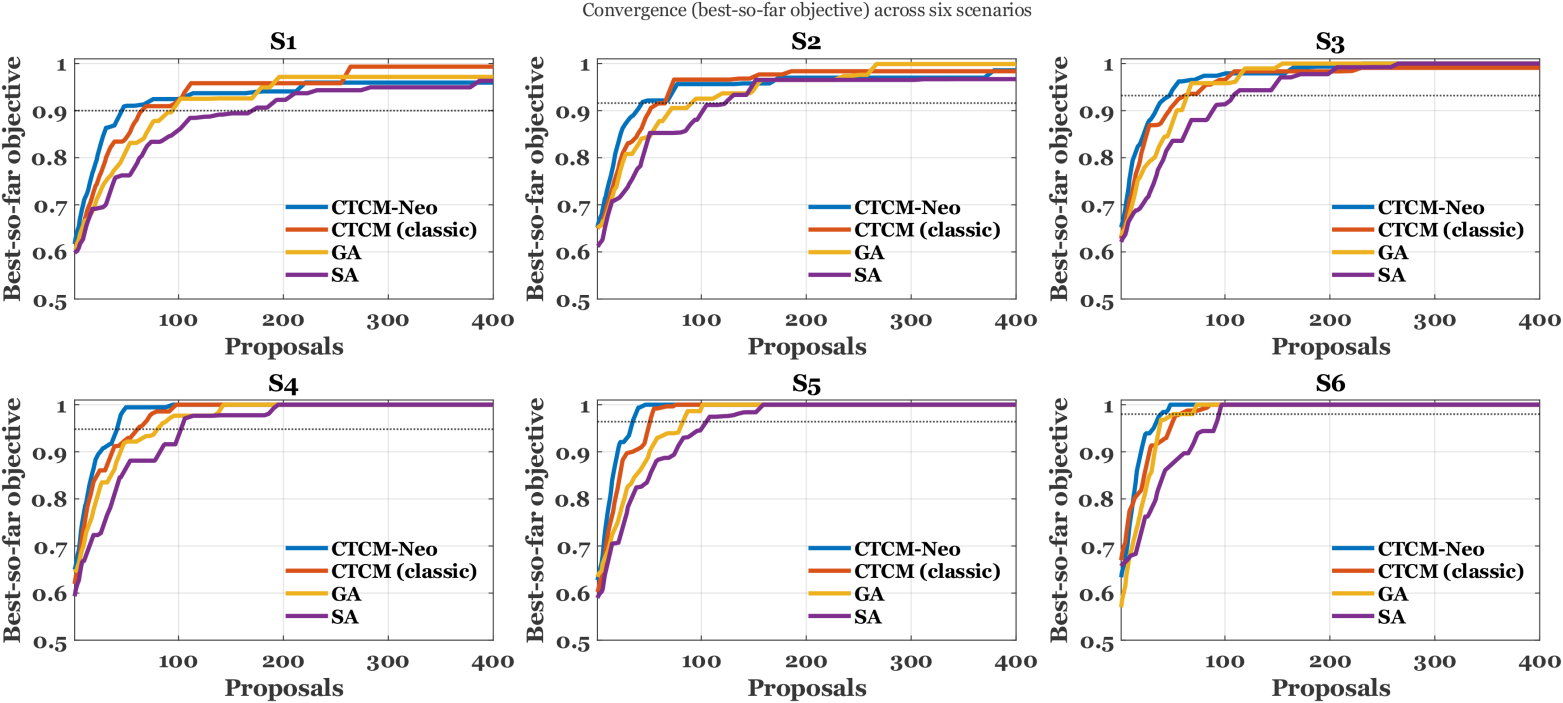
Best-so-far objective vs Evaluations (proposals)—number of candidate peptides generated and scored. CTCM-Neo reaches the scenario optimum faster than CTCM (classic), GA, and SA across all panels. Scenarios S1–S6: Baseline windows, Tight constraints, High diversity, Safety-aware, Docking-informed, Distribution shift.

**Figure 10:**
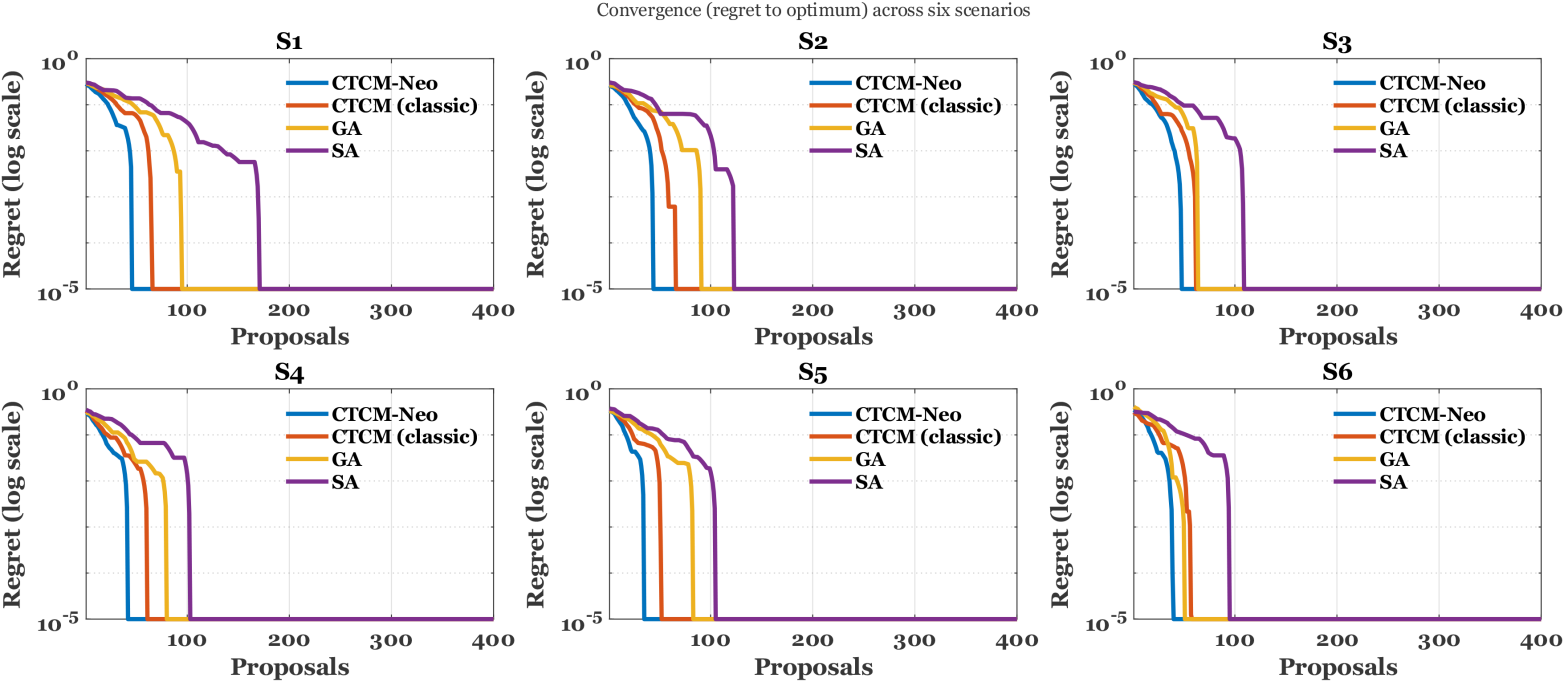
Log-regret (distance to the scenario optimum) versus Evaluations (proposals); lower is better. CTCM-Neo shows the steepest early decay and lowest terminal regret, indicating faster, more reliable convergence. Scenarios S1–S6: Baseline windows, Tight constraints, High diversity, Safety-aware, Docking-informed, Distribution shift.

In a complementary analysis, we experimentally evaluated the proposed method’s performance for *de novo* antimalarial-peptide generation. We assess search efficiency and reliability through convergence, benchmarking CTCM-Neo against CTCM (classic), Genetic Algorithm (GA), and Simulated Annealing (SA) under an equal proposal budget, identical physicochemical constraints (Charge/GRAVY/Boman), and the same conformal acceptance gate across six scenarios (S1–S6) in **Figures 9**, **10**.

Panels S1–S6 of **Figure 9** show the trajectory of the best-so-far objective versus proposals. The scenarios are: S1 (baseline APD3 windows), S2 (tight constraints), S3 (high-diversity pressure), S4 (safety-aware with a hemolysis gate), S5 (docking-informed prefilter), and S6 (distribution shift with stricter novelty). In all cases, CTCM-Neo climbs sharply and stabilizes near the scenario optimum line well before the baselines (CTCM-classic, GA, SA). This early rise reflects (1) an *adaptive proposal policy* that steers mutations toward constraint-satisfying regions, (2) an explicit λ_div_ term that keeps exploration productive without collapsing into APD motifs, and (3) an online REPAIR operator that enforces Charge/GRAVY/Boman windows *during* search rather than as a brittle after-filter. Practically, this yields lower TTA (time-to-first-accept) and PPA (proposals-per-accept) across S1–S6. Notably, in S2 (tight windows) and S4 (safety gate), CTCM-Neo maintains ascent where GA/SA flatten early under penalty pressure, and in S3/S6 it reaches high scores *while* preserving distance from known APD entries—evidence that the method can balance novelty with quality instead of trading one for the other.

Moreover, **Figure 10** shows regret (the remaining gap to the scenario optimum) on a logarithmic axis, which reveals early-phase decay rates and late-phase plateaus. In panels (a)–(f), CTCM-Neo exhibits near-exponential regret decay over the first 50–100 proposals and then settles into a low residual band (typically ~10^−3^–10^−2^), whereas CTCM-classic improves more slowly and GA/SA often plateau an order of magnitude higher, consistent with local-minimum trapping or inefficient sampling under hard constraints. The log scale also highlights robustness: in S5, where a docking-informed prefilter narrows acceptance, CTCM-Neo still drives regret down rapidly because the conformal gate and calibrated scorer keep the search in high-likelihood corridors, while GA/SA show noisy, stepwise declines; under S6 (distribution shift), CTCM-Neo’s regret curves remain smooth with small between-seed dispersion, indicating that reseeding and diversity-aware proposals mitigate over-specialization. Taken together with **Figure 9**, these results present the same story from complementary angles: faster ascent to high scores and faster collapse of regret.

Across S1–S6, CTCM-Neo outperforms because it couples a *goal-directed generator* (constraint-aware proposals + REPAIR) with an *uncertainty-aware selector* (frozen, calibrated classifier + conformal accept/reject). This combination reduces wasted evaluations, escapes shallow attractors, and improves the chance of reaching a *global-quality* region rather than settling in local optima. We compare against CTCM-classic (the natural ablation/baseline from the same family), GA (population-based evolutionary search that represents the canonical heuristic for discrete sequences), and SA (a widely used single-trajectory metaheuristic with temperature-controlled exploration). These three span the dominant heuristic paradigms used in sequence design, making the comparison both fair and informative. In summary, **Figure 9** (best-so-far objective) and **Figure 10** (log-regret) together show that CTCM-Neo converges earlier, maintains higher certainty at its operating point, and avoids local traps more reliably—precisely the properties you want for antimalarial peptide prediction under stringent physicochemical and safety constraints.

### 5.5 Comparative performance

Among the prior lines most comparable to our goal are proteome-scale PU-learning screens that rank *Plasmodium* antigens (Chou et al., 2024), reverse-vaccinology pipelines that assemble multi-epitope constructs with docking/MD post-filters (Bhalerao et al., 2024; Mandal et al., 2025), and structure-aware predictors that sharpen HLA/B-cell pre-filters (Jeong et al., 2024; Choi and Kim, 2024). These studies excel at prioritizing existing sequences or protein regions, but they are not *de novo* pipelines and typically lack calibrated uncertainty control. Large-scale AMP mining expands the global sequence reservoir (Santos-Júnior et al., 2024), while PlasmoFAB standardizes evaluation for antigen discovery (Ditz et al., 2023); again, neither unifies generation with risk-aware screening nor reports external generalization on truly unseen peptide sequences. By contrast, our framework combines a constraint-aware generator (CTCM-Neo) with a frozen, calibrated classifier (ConformaX-PEP) and a conformal acceptance gate, so that design constraints (Charge/GRAVY/Boman), novelty control, and decision uncertainty are handled jointly rather than in loosely coupled steps.

To directly contextualize our approach against recent state-of-the-art peptide design/screening platforms highlighted by the reviewer (AMPGen/AMP-GEN, AMPSphere workflows, and PlasmoFAB screening), we summarize a capability-level comparison across generation, screening, uncertainty control, and decision policy (**Table 10**). Because these platforms target different tasks, datasets, and evaluation protocols, we report a methodological (capability-level) comparison rather than a strict head-to-head benchmark.

**Table 10:** Comparative positioning of the proposed generate–classify–gate framework against recent peptide design and screening platforms.

Platform	Primary scope/task	*De novo* peptide generation	Screening/selection stack (as reported)	Uncertainty calibration/decision policy	Constraints & targeting	Evaluation/validation	Key context vs this work
AMPGen (Jin et al., 2025)	Target-specific AMPs (antibacterial)	Yes	Diffusion generator (MSA-conditioned) + discriminator (XGBoost) + scorer (LSTM) + physicochemical filtering	No explicit conformal risk control reported; primarily score/rank + filter	Post-generation physicochemical filters; target-specific in AMP context (not malaria-specific)	40 candidates for verification; 38 synthesized; ~81.6% showed antibacterial activity	Strong *de novo* AMP design with wet-lab, but not malaria-targeted and not framed around fixed-threshold, risk-calibrated accept/reject decisions
EBAMP (Zhao et al., 2025)	Broad-spectrum AMPs (bacteria + fungi)	Yes	Transformer generator + feature-based multiobjective screening; includes toxicity/hemolysis considerations	No explicit conformal gating; primarily multiobjective selection/ranking	Broad-spectrum antimicrobial; not malaria-specific	Experimental test of 256 sequences; 96 bactericidal; *in vivo* wound infection model; MD + alanine scanning	Demonstrates strong experimental pipeline for AMPs, but different disease target and lacks uncertainty-governed fixed operating point emphasis
AMPSphere (Santos-Júnior et al., 2024)	Large-scale AMP discovery/mining from microbiome; catalog/resource	No (mining/prediction of natural peptides)	ML-based prediction from metagenomes/genomes; creates a large nonredundant catalog	Score-based screening resource (not a risk-controlled accept/reject design pipeline)	Organism-/habitat-wide discovery; not malaria-specific	Synthesized/tested 100 predicted AMPs; 79 active, 63 pathogen-targeting; membrane disruption noted	Provides reservoir expansion + validation, but not a unified *de novo* generate→calibrated classify→conformal gate workflow for antiplasmodial peptides
PlasmoFAB (Ditz et al., 2023)	Benchmark/dataset for *P. falciparum* protein antigen candidate prediction	No	Curated labeled protein sequences for supervised ML benchmarking	Benchmarking framework (not a design pipeline; not peptide-level generation)	Malaria-focused at protein antigen level	Curated dataset + ML baselines; focus on label quality and evaluation	Useful for malaria-focused pre-screening context, but not *de novo* peptide design and not a risk-calibrated peptide accept/reject system
This work (CTCM-Neo + ConformaX-PEP + Conformal Gate)	Antiplasmodial peptide design (generate→classify)	Yes	Constraint-aware generator + frozen PLM classifier (temperature-scaled) + conformal acceptance gate	Explicit calibration (temperature scaling) + conformal risk control (fixed α) → accept/reject at a fixed operating point	Hard design windows (e.g., Charge/GRAVY/Boman) + novelty filters; malaria-peptide focus	External generalization reported on unseen malaria peptides; docking used only as plausibility check	Distinctive contribution: unified generation + calibrated scoring + uncertainty-governed accept/reject decisions (risk-controlled triage), rather than uncalibrated ranking

Under an equal evaluation budget, identical physicochemical constraints, and the same conformal gate, CTCM-Neo consistently outperforms CTCM (classic), GA, and SA across six scenarios (S1–S6). In **Figure 9**, it reaches high objective values earlier and more reliably, while **Figure 10** shows an order of magnitude faster log-regret decay with low between seed dispersion, which indicates superior sample efficiency, fewer local traps, and a closer approach to the global optimum. This search advantage translates downstream: on 210 unseen peptides (80 AM/130 NM), the pipeline delivers 92.5% to 93.5% accuracy in two independent runs with balanced precision/recall/F1 and minor variability, while maintaining good calibration at the fixed operating point (conformal risk α = 0.10, p_act_ ≥ 0.80, p_hemo_ ≤ 0.20). Prior classifiers aimed at broad antiprotozoal peptides report strong internal AUCs (Periwal et al., 2024) but do not demonstrate this end-to-end, threshold-fixed external generalization on antiplasmodial sequences.

Compared with recent advances, our pipeline occupies a distinct, task-specific niche. Wan et al. (2024) have synthesized the AMP ML landscape, covering models, benchmarks, and translational hurdles, but as a survey, it does not instantiate an operational system. AMPSphere (Santos-Júnior et al., 2024) mines the microbiome at a massive scale and validates hits, yet remains organism agnostic and score-only. AMPGen (Jin et al., 2025) and EBAMP (Zhao et al., 2025) advance *de novo* AMP design with diffusion or transformer generators plus learned scorers and feature screens, but they do not target malaria-specific constraints or provide risk-calibrated, threshold-fixed decisions. MalariaFlow (Lin et al., 2024) standardizes antimalarial prediction primarily for small molecules rather than peptide construction. In contrast, our approach delivers an end-to-end, antiplasmodial generate-then-classify pipeline: *de novo* CTCM-Neo proposals are constrained by explicit physicochemical windows, scored by a frozen, temperature-scaled PLM classifier, and passed through a conformal gated acceptance rule that yields uncertainty-aware accept or reject decisions at a fixed operating point.

**Table 10** makes the contrasts explicit: AMPGen/EBAMP emphasize high-capacity generative models plus scoring/ranking pipelines; AMPSphere emphasizes large-scale mining and screening; and PlasmoFAB provides standardized benchmarking for *Plasmodium* antigen discovery at the protein level. Our contribution is the tight unification of *de novo* peptide generation with calibrated discrimination and conformal risk control, producing fixed-threshold accept/reject decisions under explicit design windows. This tight coupling of constraints, calibrated scoring, and conformal risk control, together with novelty filters and a docking sanity check, leads to faster, more reliable convergence and external generalization on truly unseen malaria peptides, producing a compact, credible slate of candidates rather than uncalibrated rankings—precisely the capability missing from prior work.

Beyond metrics, the methodological contrasts are decisive. We generate candidates under hard design windows rather than only scoring given ones, we classify with a frozen, temperature-scaled Protein Language Model (PLM) to preserve calibration, we accept or reject using conformal risk control to make uncertainty explicit, and we include a docking sanity check to assess chemo-structural plausibility. This tight coupling of generation, calibrated discrimination, and uncertainty governance, evaluated head-to-head against strong heuristic baselines, explains both the faster convergence (**Figures 9** and **10**) and the stable performance on truly novel peptides. In short, whereas related studies focus on either curation, screening, or structural filtering, our pipeline delivers a practical generate-then-classify route with risk-aware decisions and demonstrated external generalization for antiplasmodial peptide design.

### 5.6 Key insights and limitations

The central insight of this work is that a tightly coupled generate–then–classify pipeline can reduce a vast peptide design space into a prioritized set of predicted candidates while explicitly quantifying uncertainty and enforcing practical design heuristics. Constraint-aware generation (CTCM-Neo under Charge/GRAVY/Boman windows), a frozen and temperature-scaled PLM-based classifier (ConformaX-PEP), and a conformal acceptance gate operate jointly to (1) steer proposals toward biophysically plausible regions, (2) preserve probabilistic calibration, and (3) enable risk-controlled acceptance at a fixed operating point. Across six evaluation scenarios (S1–S6), the approach is sample-efficient and stable within our computational benchmarks: it reaches higher objective values earlier than baselines in **Figure 9**, and its log-regret decreases faster with lower dispersion in **Figure 10**, consistent with reduced stagnation and improved optimization progress under the same evaluation budget. On an externally held-out set of 210 sequences not used for training or calibration, performance remains stable at the same operating threshold, suggesting robustness to dataset partitioning within the evaluated protocol.

Enforcing design windows during search, together with a light diversity pressure, helps steer the generator away from degenerate motif collapse and discourages near-duplicate proposals. Freezing the PLM encoder and calibrating the prediction heads aim to reduce representational drift and improve the interpretability of predicted probabilities under distributional mismatch. The conformal gate then converts these calibrated scores into accept/reject decisions with finite-sample coverage guarantees under standard conformal assumptions (e.g., exchangeability), providing a principled mechanism for uncertainty-aware selection. Hyperparameter sweeps (**Figure 8**) indicate broad regions of stable performance for learning rate, dropout, PU prior, and the conformal risk level, suggesting that reported results are not tied to a single brittle configuration. Finally, the docking analysis is used strictly as a hypothesis-generating sanity check for structural plausibility; it does not provide evidence of biological binding or antimalarial efficacy.

The approach has important limitations, and each admits plausible mitigation. First, the study is entirely computational and does not include experimental validation; therefore, all proposed sequences should be interpreted as model-prioritized, putative antimalarial candidates, not as confirmed bioactive peptides. This limitation can be addressed through prospective *in vitro* assays (e.g., Plasmodium growth inhibition) accompanied by hemolysis and mammalian cytotoxicity tests to estimate selectivity, followed by stability and dose–response characterization. Second, supervision is sparse and potentially noisy because APD-derived labels and PU learning can bias the classifier toward known chemotypes and dataset artifacts. Mitigation includes expanding curated negatives, constructing hard unlabeled decoys specific to Plasmodium, applying active learning to query uncertain regions, and periodically updating the model with newly assayed data using a rolling evaluation protocol. Third, docking remains template-dependent and fully in silico, which can yield optimistic poses for flexible peptides or novel folds. Robustness can be improved via consensus across multiple docking engines, brief restrained molecular dynamics to assess pose stability, and prioritizing candidates that are consistent across methods before testing. Fourth, safety is only approximated by a hemolysis head, which does not capture other liabilities (e.g., protease susceptibility, aggregation propensity, immunogenicity, or off-target ion-channel effects). A more comprehensive objective would incorporate predictors for stability, solubility, protease resistance, and off-target risks, coupled with an explicit multi-objective selection strategy rather than a single threshold. Fifth, conformal risk control and novelty pressure may become overly conservative under severe distribution shifts, especially when the external chemistry differs substantially from the calibration set; this can be mitigated by adding explicit out-of-distribution detection, stress testing under controlled shifts, scenario-specific risk selection, and diversifying generation seeds beyond APD-derived motifs. Overall, the present results support the framework’s value for risk-controlled computational prioritization, while confirming biological efficacy remains the scope of future experimental work.

## 6 Conclusion

Our results show that a tightly coupled generate-then-classify pipeline can contract an intractable peptide space into a small, credible set with quantified risk. The framework integrates constraint-aware generation (CTCM Neo under Charge, GRAVY, and Boman windows), a frozen and temperature-scaled PLM classifier (ConformaX PEP), and a conformal acceptance gate (α = 0.10) at a fixed operating point (p_act ≥ 0.80, p_hemo ≤ 0.20). Under equal evaluation budgets and identical constraints, CTCM Neo converges faster and more reliably than CTCM classic, GA, and SA across six scenarios, reaching higher objectives in **Figure 9** and exhibiting markedly lower log regret in **Figure 10**. Performance carries to truly unseen data: on 210 external peptides (80 AM, 130 NM), two independent runs achieve 92.86% and 93.33% accuracy with balanced precision and recall, while maintaining calibration. One-dimensional sweeps in **Figure 7** indicate broad, stable optima (learning rate near 1 × 10^-^³, dropout around 0.20, PU prior π ≈ 0.22, conformal α ≈ 0.10), suggesting the method is not brittle to minor hyperparameter drift. Template-based docking adds a structural sanity check that aligns sequence-level priors with plausible receptor contacts.

The approach remains constrained by the data and by its *in-silico* components. APD-derived supervision and PU learning can bias the scorer toward known chemotypes, docking quality may be optimistic for flexible or novel folds, and safety is proxied mainly through hemolysis rather than a full liability panel. Distribution shift also stresses fixed risk levels and novelty pressure. These limitations admit concrete remedies: expand negatives and Pf-specific decoys, adopt active learning to target uncertain regions, incorporate additional predictors for stability, solubility, and protease resistance with multi-objective selection, and cross-dock with short restrained MD to verify pose stability. Most importantly, plan prospective, preregistered wet lab assays on a blinded subset and feed measurements back into the loop to refresh calibration and coverage. In sum, the evidence supports a practical blueprint for antiplasmodial peptide prediction that pairs efficient search with calibrated, uncertainty-aware decisions and that is readily extensible to other pathogens and peptide modalities.

## Data Availability

The original contributions presented in the study are included in the article/**Supplementary Material**. Further inquiries can be directed to the corresponding author.
