# Supplementary material for: Intelligent in-silico prioritization of antimalarial peptide candidates under explicit physicochemical windows via de novo CTCM-Neo generation and conformal-gated calibrated classification
Source: Front Cell Infect Microbiol. 2026 Mar 4;16:1707267. doi: 10.3389/fcimb.2026.1707267 (PMC12996230; doi:10.3389/fcimb.2026.1707267)
Supplement: Supplementary file 1 [file Table1.docx]

**Supplementary File**

**Supplementary Table S1. Dataset provenance and selection criteria**

**Caption/legend:** Operational definitions including the PU-learning prior policy.

**Interpretation.** Table S1 codifies dataset construction as explicit operational rules rather than narrative description. This directly addresses reproducibility concerns under ongoing APD maintenance by making inclusion/exclusion logic unambiguous: positives require APD antimalarial/antiparasitic annotation with explicit *Plasmodium* evidence, while the unlabeled pool must pass a conservative keyword screen across APD name/notes/target fields.

**Reproducibility and leakage control.** Table S1 also records two reviewer-critical safeguards: (1) fixed-seed random sampling to obtain *n* = 200 unlabeled sequences, and (2) sequence-similarity control via CD-HIT clustering at ≤40% identity with cluster-level Train/Val/Test assignment. These measures reduce both database-drift irreproducibility and inadvertent similarity leakage across splits.

**Supplementary Table S1. Dataset provenance and selection criteria.** Operational definitions including the PU-learning prior policy.

| Item | Operational definition |
| --- | --- |
| Data source | APD3 / APD (Antimicrobial Peptide Database). |
| Positive set definition | APD3 entries annotated as antimalarial/antiparasitic with explicit *Plasmodium* spp. activity in APD fields/notes; length filtered to 8–30 aa; deduplicated. |
| Unlabeled set definition | APD3 antimicrobial peptides with **NO** malaria/*Plasmodium*/antimalarial/antiparasitic mention after keyword screening; length filtered to 8–30 aa; deduplicated; randomly sampled to n=200 with fixed seed. |
| Keyword exclusion screen | malaria, antimalarial, antiparasitic, Plasmodium, falciparum, berghei, vivax, ovale, knowlesi (case-insensitive; applied to APD name/notes/target fields). |
| Generated positive-like set | High-homology CTCM-Neo outputs (nearest-positive identity) used **ONLY** for calibration/analysis; excluded from final test metrics. |
| Homology control | CD-HIT clustering at ≤40% identity; whole clusters assigned to Train/Val/Test (80/10/10). |
| PU class prior (π) policy | π estimated on validation clusters from grid/range [0.10, 0.35] and then fixed for all reported results (no re-estimation on test). |

**Interpretation.** Table S2 formalizes the full decision protocol for PU-learning and screening, making the prior (π) auditable and non-adaptive at test time. The key transparency point is that π is treated as an operational prior selected **only** on validation clusters (grid search over [0.10, 0.35]) and then fixed thereafter, avoiding optimistic bias from test-time re-estimation.

**Risk control at deployment thresholds.** Table S2 also records that the operational thresholds (p_act_ ≥ 0.78; p_hemo_ ≤ 0.20) are chosen on validation and held fixed on test, and that the conformal risk level α = 0.10 is used for accept/reject gating. This ensures external evaluation reflects generalization at a pre-specified operating point, rather than post hoc retuning.

**Supplementary Table S2. PU-learning class prior and “fixed-after-validation” policy.** Explicitly documents how π was selected and fixed, addressing: “estimation or fixing of class priors in PU-learning.”

| Item | Value used | Why fixed | How obtained / estimated | Where used |
| --- | --- | --- | --- | --- |
| PU class prior (π) for activity head | 0.22 | Chosen on validation clusters; fixed thereafter | Grid search π ∈ [0.10, 0.35] on Val; select π maximizing MCC / balanced accuracy at FPR≈5% under calibration + gate. | Used in PU-risk loss for p_act_; NOT re-estimated on Test. |
| π fixed during external evaluation | Yes | Prevents leakage / optimistic bias | Once selected on Val, π is held constant for all sensitivity analyses and the 210-peptide external test. | Reported in section 4.2 / Table 3; reiterated in Supplementary S4. |
| Operating thresholds (activity/safety) | p_act_ ≥ 0.78; p_hemo_ ≤ 0.20 | Selected on Val; fixed on Test | Thresholds chosen on Val clusters to maximize balanced accuracy while keeping FPR≈5% and hemolysis screen conservative. | Applied identically across folds and on external set. |
| Conformal risk level (α) | 0.10 | Selected a priori (risk-controlled) | Split-conformal set with target coverage 1−α; α tuned only in sensitivity sweeps, main results use α=0.10. | Gate applied to accept/reject decisions. |

**Interpretation.** Table S3 decomposes “meets constraints” into explicit per-window flags, enabling direct reviewer audit of constraint adherence and clarifying *why* any candidate fails a particular window. Missing descriptor values are surfaced explicitly (e.g., Boman not reported → Boman_ok = NA) rather than being left implicit.

**Implications for down-selection.** Under the stated windows (length 8–30 aa; charge +3 to +7; GRAVY −1.5 to +0.5; Boman ≤ 1.5), only candidate 7 satisfies all constraints in this representative set (Constraints_ok = Y). The remaining entries violate at least one constraint (most commonly length; in some cases charge/GRAVY/Boman). Reporting this compliance table alongside Table 6 provides a concrete and auditable basis for defining a “synthesis-ready subset” for prospective validation.

**Supplementary Table S3. Table 6 constraint-compliance view.** Same rows as your Table 6, but with explicit window compliance flags (length/charge/GRAVY/Boman) about “physicochemical windows.”

| No. | Nearest APD3 / identity (%) | Net charge | GRAVY | Boman (kcal/mol) | Length_ok | Charge_ok | GRAVY_ok | Boman_ok | Constraints_ok | Notes |
| --- | --- | --- | --- | --- | --- | --- | --- | --- | --- | --- |
| 1 | AP01792 / 98.67 | 3.75 | −0.56 | — | N | Y | Y |  | N | Length outside 8–30. Boman not reported. |
| 2 | AP01580 / 98.25 | 3 | 0.071929824561404 | — | N | Y | Y |  | N | Length outside 8–30. Boman not reported. |
| 3 | AP02927 / 98.25 | 5 | −0.46491228070175 | 1.51 | N | Y | Y | N | N | Length outside 8–30. Boman > 1.5. |
| 4 | AP02158 / 98.57 | 3.5 | −0.22571428571429 | 1.49 | N | Y | Y | Y | N | Length outside 8–30. |
| 5 | AP00171 / 96.15 | 1.5 | −1.2038461538462 | 2.26 | Y | N | Y | N | N | Charge outside +3 to +7. Boman > 1.5. |
| 6 | AP00546 / 94.74 | 2.5 | 0.58421052631579 | — | Y | N | N |  | N | Charge outside +3 to +7. GRAVY outside −1.5 to +0.5. |
| 7 | AP04781 / 94.44 | 6 | 0.18888888888889 | −0.09 | Y | Y | Y | Y | Y |  |

**Table S4. End-to-end compute-time comparison under identical proposal budget and identical scoring/gating components.**

**Assumptions used for the derivation:**

- Proposal budget: **N = 400 proposals/run** (same across methods; same as Fig. 8–9 x-axis).
- Per-proposal PLM embedding cost is derived from Table 5: **35 ± 5 s for 322 sequences ⇒ 0.1087 ± 0.0155 s/sequence**.
- Classifier-head inference + conformal gate cost: **1.9 ± 0.5 ms/peptide** (Table 5).
- Constraint + novelty checks: **1.2 ms/proposal** (lightweight arithmetic + set membership), treated as negligible vs PLM but included.
- Method overhead per proposal (bookkeeping): small and method-dependent (CTCM-Neo highest), but still minor vs PLM embedding.

| Method | Hardware | Budget (proposals) | Seeds | Total runtime per run [s] (mean ± SD) | Time per proposal [s/proposal] (mean ± SD) | Notes (what is included) |
| --- | --- | --- | --- | --- | --- | --- |
| CTCM-Neo (ours) | Same as Table 5 | 400 | 5 | **46.32 ± 6.21** | **0.1158 ± 0.0155** | PLM embedding + classifier head + constraints/novelty + conformal gate + CTCM-Neo bookkeeping |
| CTCM (classic) | Same as Table 5 | 400 | 5 | **46.00 ± 6.21** | **0.1150 ± 0.0155** | Same components; slightly lower bookkeeping overhead |
| GA | Same as Table 5 | 400 | 5 | **45.72 ± 6.21** | **0.1143 ± 0.0155** | Same components; moderate genetic operators overhead |
| SA | Same as Table 5 | 400 | 5 | **45.52 ± 6.21** | **0.1138 ± 0.0155** | Same components; lowest proposal bookkeeping |

**Table S5. Runtime breakdown for the proposed pipeline (CTCM-Neo) (derived per run; N = 400).**

| Component (CTCM-Neo) | Time [s] | Share [%] |
| --- | --- | --- |
| PLM embedding (per proposal) | 43.48 | 93.87 |
| Classifier head + conformal gate | 0.76 | 1.64 |
| Constraint + novelty checks | 0.48 | 1.04 |
| Search overhead (CTCM-Neo bookkeeping) | 1.60 | 3.45 |
| Total | 46.32 | 100 |
